# Loss of caveolar A_1_ adenosine receptor signalling blunts anti-adrenergic control in heart failure

**DOI:** 10.1093/cvr/cvag131

**Published:** 2026-06-24

**Authors:** Marta Mazzola, Houcheng Wang, Di Lang, Marina Balycheva, Navneet Bhogal, Jose L Sanchez-Alonso, Ivan Diakonov, Carla Lucarelli, En-Chi Lai, Prakash P Punjabi, Michele Miragoli, Giuseppe Faggian, Alexey V Glukhov, Julia Gorelik

**Affiliations:** National Heart and Lung Institute, Imperial College London, Imperial Centre for Translational and Experimental Medicine, Hammersmith Campus, Du Cane Road, London W12 0NN, UK; Department of Surgery, Dentistry, Pediatrics and Gynecology, Devision of Cardiac Surgery, University of Verona, 37129, Verona, Italy; National Heart and Lung Institute, Imperial College London, Imperial Centre for Translational and Experimental Medicine, Hammersmith Campus, Du Cane Road, London W12 0NN, UK; Department of Medicine, School of Medicine and Public Health, University of Wisconsin-Madison, Madison, 1111 Highland Ave 8455 WIMR2, Madison, WI 53705, USA; Department of Medicine, University of California San Francisco, 555 Mission Bay Boulevard South, San Francisco, CA, USA; National Heart and Lung Institute, Imperial College London, Imperial Centre for Translational and Experimental Medicine, Hammersmith Campus, Du Cane Road, London W12 0NN, UK; Department of Surgery, Dentistry, Pediatrics and Gynecology, Devision of Cardiac Surgery, University of Verona, 37129, Verona, Italy; Department of Medicine, School of Medicine and Public Health, University of Wisconsin-Madison, Madison, 1111 Highland Ave 8455 WIMR2, Madison, WI 53705, USA; National Heart and Lung Institute, Imperial College London, Imperial Centre for Translational and Experimental Medicine, Hammersmith Campus, Du Cane Road, London W12 0NN, UK; National Heart and Lung Institute, Imperial College London, Imperial Centre for Translational and Experimental Medicine, Hammersmith Campus, Du Cane Road, London W12 0NN, UK; National Heart and Lung Institute, Imperial College London, Imperial Centre for Translational and Experimental Medicine, Hammersmith Campus, Du Cane Road, London W12 0NN, UK; National Heart and Lung Institute, Imperial College London, Imperial Centre for Translational and Experimental Medicine, Hammersmith Campus, Du Cane Road, London W12 0NN, UK; National Heart and Lung Institute, Imperial College London, Imperial Centre for Translational and Experimental Medicine, Hammersmith Campus, Du Cane Road, London W12 0NN, UK; National Heart and Lung Institute, Imperial College London, Imperial Centre for Translational and Experimental Medicine, Hammersmith Campus, Du Cane Road, London W12 0NN, UK; Department of Cardiothoracic Surgery, Hammersmith Hospital, National Heart and Lung Institute, Imperial College London, Hammersmith Campus, Du Cane Road, London W12 0NN, UK; Department of Medicine and Surgery, University of Parma, Via Gramsci, 14 - 43124, Parma, Italy; IRCCS- Humanitas Research Hospital, Rozzano, Via Alessandro Manzoni, 56, 20089, Rozzano, Milan, Italy; Department of Surgery, Dentistry, Pediatrics and Gynecology, Devision of Cardiac Surgery, University of Verona, 37129, Verona, Italy; Department of Medicine, School of Medicine and Public Health, University of Wisconsin-Madison, Madison, 1111 Highland Ave 8455 WIMR2, Madison, WI 53705, USA; National Heart and Lung Institute, Imperial College London, Imperial Centre for Translational and Experimental Medicine, Hammersmith Campus, Du Cane Road, London W12 0NN, UK

**Keywords:** Adenosine A1 receptor, Β_1_-adrenergic receptor, T-tubules, Caveolae, Heart failure, Optical mapping, FRET microscopy

## Abstract

**Aims:**

Adenosine, acting through A_1_ adenosine receptors (A_1_ARs), exerts anti-adrenergic effects by inhibiting β_1_-adrenergic receptor (β_1_AR)–mediated cyclic adenosine monophosphate (cAMP) production and contractility in the heart. While the functional interaction between A_1_ARs and β_1_ARs is well established in both atrial and ventricular myocytes, the subcellular compartmentalization of this crosstalk and how it is disrupted in heart failure (HF) remains incompletely understood. This study investigates the spatial confinement of A_1_AR–β_1_AR signalling within atrial microdomains and assesses how structural remodelling in HF alters this regulatory axis.

**Methods and results:**

Quantitative polymerase chain reaction (qPCR) analysis revealed that A_1_AR is the predominant adenosine receptor subtype in both rat and human atrial tissues. In healthy rat and mouse atrial myocytes, A_1_AR activation reduced β_1_AR-induced cAMP production and sarcomere shortening, with suppression of cAMP signals at sarcolemmal microdomains enriched in protein kinase A Type II. This was further supported by scanning ion conductance microscopy–guided scanning patch-clamp, which showed that A_1_AR suppressed β_1_AR-driven L-type Ca^2+^ channel activity at both T-tubule and crest membrane domains. In atrial myocytes isolated from failing rat and human hearts, A_1_AR-mediated inhibition of β_1_AR-induced cAMP production and contractility was impaired. Caveolar disruption by methyl-β-cyclodextrin in rat atrial myocytes or via cardiac-specific caveolin-3 (Cav3) knockout in mice abolished this A_1_AR-mediated inhibition. Notably, cholesterol repletion alone did not restore membrane cAMP regulation, whereas Cav3 overexpression rescued A_1_AR-dependent suppression, supporting a requirement for Cav3-dependent organization. In mouse atrial preparations isolated from failing hearts, high-resolution optical mapping showed that A_1_AR-mediated anti-adrenergic regulation of Ca^2+^ cycling was selectively lost in the intercaval region, correlating with the regional absence of T-tubule and downregulation of caveolae structures.

**Conclusion:**

A_1_ARs provide anti-adrenergic restraint of β_1_AR signalling through Cav3-dependent membrane organization. In HF, regional caveolar disorganization uncouples this protective pathway, contributing to spatially heterogeneous Ca^2+^ dysregulation in the atrium.

**Time for primary review: 32 days**

## 1. Introduction

Precise spatial and temporal regulation of cyclic adenosine monophosphate (cAMP) signalling is critical for maintaining coordinated excitation–contraction (EC) coupling in the heart through various neuro-hormonal signalling pathways.^1^ cAMP provides a crucial coupling between sarcolemmal G protein–coupled receptors and subcellular components of Ca^2+^ handling machinery and contractile apparatus, mainly via activation of protein kinase A (PKA) and subsequent phosphorylation of L-type Ca^2+^ channels (LTCCs), ryanodine receptors, phospholamban, and Troponin I. β_1_-Adrenergic receptors (β_1_ARs), which couple to G_s_ proteins and thus stimulate cAMP production, are key regulators of EC-coupling.^2,3^ The activation of this signalling cascade enhances cardiac inotropy in response to sympathetic regulation of the heart.

Adenosine is an endogenous metabolite of the heart^4^ and exerts various cardioprotective effects directed at optimizing the balance between energy utilization and generation through fine-tuning of adrenergic sensitivity on the heart. One of the major cardioprotective effects of adenosine involves modulation of cardiac contractility, primarily in an indirect fashion, through the attenuation of the positive inotropic effect of βAR stimulation and is believed to serve as a negative feedback modulator of catecholamine-elicited responses in the heart. The anti-adrenergic effects of adenosine are primarily mediated through the A_1_ adenosine receptor (A_1_AR), which couples to G_i_ proteins to inhibit adenylyl cyclase activity and reduce intracellular cAMP levels. While the ability of A_1_ARs to oppose β_1_AR-driven signalling has been well established, in both atrial and ventricular myocardium, the subcellular organization coupling of A_1_AR, and how this is disrupted in disease, is incompletely understood.

In cardiomyocytes, compartmentation of G protein–coupled receptors signalling is enabled by the formation of membrane microdomains, including caveolae—flask-shaped invaginations of the sarcolemma enriched in cholesterol and scaffolded by caveolin-3 (Cav3).^5,6^ These domains restrict the diffusion of receptors, G proteins, phosphodiesterase, and ion channels, thereby supporting highly localized signalling.^7–10^ A_1_AR signalling has been linked to caveolae nanodomains^11^ via the direct interaction between the cytoplasmic C-terminal domain of A_1_ARs with Cav-1 scaffolding domain,^12^ which is structurally similar to scaffolding domain of cardiac-specific Cav3.^13^ Importantly, a substantial proportion of atrial LTCCs have also been associated with caveolae nanodomains, especially in cells lacking developed T-tubular system.^14^ Furthermore, recent studies also linked cAMP signalling to caveolae structures, highlighting their key role in organizing macromolecular complexes and providing structural foundation for G protein–coupled receptors/cAMP signalling.^15^

Heart failure (HF) induces structural remodelling that disrupts this spatial architecture with elevated extracellular adenosine levels and impaired adenosine receptor function.^16^ Although Cav3 expression is often preserved, caveolar flattening and disorganization are common, and even the sparse T-tubule network in atrial cells becomes further depleted and disordered.^7,17^ These alterations may disrupt the integrity of receptor–effector coupling within the sarcolemma. Whether such remodelling leads to functional uncoupling of A_1_ARs from downstream G_i_-cAMP signalling and LTCC inhibition remains unknown.

In this study, we investigated the spatial regulation of A_1_AR signalling in healthy and failing rat, mouse, and human atrial myocytes using a cutting-edge combination of super-resolution scanning patch-clamp electrophysiology, Fluorescence resonance energy transfer (FRET)-based cAMP imaging, high-resolution topographical mapping, and fluorescent optical mapping of Ca^2+^ transients from *ex vivo* atrial preparations and isolated atrial cardiomyocytes. We show that A_1_AR activation restrains β_1_AR-driven cAMP signalling and LTCC activity within Cav3-dependent membrane domains that support EC-coupling. A key role of Cav3 in structural and functional organization of A_1_AR-blunted signalling is further supported by experiments showing that Cav3 overexpression restores membrane-local A₁AR suppression of β₁AR-driven cAMP after caveolar disruption, whereas cholesterol repletion alone is insufficient and dominant-negative Cav3 (Cav3DN) fails to restore the response. In failing human and rat cardiomyocytes, A_1_AR-mediated inhibition of β_1_AR-driven cAMP and contractility is significantly reduced despite preserved A_1_AR and Cav3 expression. At the tissue level, optical mapping shows that A_1_AR anti-adrenergic regulation of Ca^2+^ handling is preserved in the right atrial appendage (RAA) but selectively lost in the intercaval region (ICR) in failing mice, and transmission electron microscopy (TEM) demonstrates a corresponding regional reduction in caveolae density. Collectively, these findings identify membrane microdomain integrity as a determinant of endogenous adenosine protection in the atrium and suggest that preserving or restoring Cav3-dependent organization may help re-establish anti-adrenergic control in HF-associated atrial dysfunction.

## 2. Methods

### 2.1 Experimental model and subject details

All animal studies were approved by the Animal Welfare and Ethics Review Board at Imperial College London and the University of Wisconsin–Madison and were performed in accordance with the UK Animals (Scientific Procedures) Act 1986, EU Directive 2010/63/EU, and the NIH Guide for the Care and Use of Laboratory Animals (NIH publication No. 85-23, revised 1996). Humane care was ensured throughout. For terminal tissue collection and cardiomyocyte isolation, rats were euthanized by cervical dislocation following sedation with 5% isoflurane. Human atrial tissue collection was approved by the institutional review boards of Imperial College London and the University of Verona. All investigations involving human tissue were conducted in accordance with the principles outlined in the Declaration of Helsinki, and informed written consent was obtained prior to inclusion in the study.

### 2.2 Mouse lines and breeding

#### 2.2.1 Cav3 knockout mice

To achieve cardiomyocyte-specific deletion of Cav3, mice harbouring loxP-flanked Exon 2 of the Cav3 gene were crossed with transgenic mice expressing tamoxifen-inducible Cre recombinase under control of the α-myosin heavy chain promoter (α-MHC-MerCreMer), as previously described.^15^

Tamoxifen was administered via the diet at 0.5 mg/g. Powdered chow was reconstituted in distilled water (1 mL/g), reformed into pellets, and dried at room temperature for 24–48 h. Mice were maintained on this diet for 14 consecutive days. Experiments were conducted 14 days after completing tamoxifen treatment to allow for gene recombination and protein depletion.^18^ Genotyping was performed by polymerase chain reaction (PCR) on genomic DNA extracted from ear or tail biopsies. Colonies were maintained as homozygous lines.

#### 2.2.2 CAG-Epac1-camps mice

To monitor cytosolic cAMP levels, we used transgenic CAG-Epac1-camps mice, which ubiquitously express a FRET-based biosensor composed of the Epac1 cAMP-binding domain flanked by cyan fluorescent protein (CFP) and yellow fluorescent protein (YFP) fluorophores. The sensor is driven by the cytomegalovirus (CMV) early enhancer/chicken β-actin) CAG promoter, a hybrid cytomegalovirus enhancer and chicken β-actin promoter.^19^ Mice were provided by Professor V.O. Nikolaev (University Medical Center Hamburg-Eppendorf, Germany). Mice were genotyped by PCR and screened for sensor expression using fluorescence goggles. Homozygous animals were used in all experiments involving cAMP imaging.^20^

#### 2.2.3 Generation of double transgenic mice (CAG-Epac1-camps/Cav3 knockout)

To assess the impact of caveolae on compartmentalized cAMP signalling, homozygous CAG-Epac1-camps mice were crossed with Cav3 knockout (Cav3KO) mice. Offspring were genotyped to confirm presence of both transgenes and screened for sensor fluorescence. Successive filial crosses were performed until stable homozygosity for both the Epac1 sensor and conditional Cav3KO alleles was achieved. PCR and green fluorescent protein (GFP) miners goggles (Biological Laboratory Equipment, Maintenance & Service Ltd) were used for the screening.

### 2.3 Rat and mouse HF model

Sixteen weeks post-myocardial infarction (MI) rats were generated as previously described.^14,21,22^ Briefly, the adult Sprague-Dawley rats (weighing 250 g) were anaesthetized with isoflurane (5% reduced to 2% once intubated and ventilated) and were given the antibiotic enrofloxacin (5 mg/kg) and 0.9% saline (10 mL/kg) as well as buprenorphine subcutaneously (0.05 mg/kg) for pain relief. After anaesthesia, a suture was made around the left anterior descending (LAD) coronary artery to bound and constrict the blood flow with 6-0 silk. Cardiomyocytes were isolated after sixteen weeks post-MI.

Eight weeks post-MI, HF was induced in male C57BL/6J mice (University of Wisconsin–Madison Cardiovascular Physiology Core Facility) by permanent ligation of the LAD coronary artery, as previously described.^23^ Mice (8–10 weeks old) were anaesthetized with 2% isoflurane in oxygen and mechanically ventilated. A left thoracotomy was performed to expose the heart, and the LAD artery was ligated using an 8-0 nylon suture. Successful induction of acute MI was confirmed by visual blanching of the left ventricular (LV) free wall. Mice were maintained on a heating pad during recovery until fully awake. HF progression was assessed 8 weeks post-MI using transthoracic echocardiography to evaluate LV structure and function.

### 2.4 Measurement of cardiomyocyte contractility

Atrial cardiomyocytes contraction was assessed by determining cell shortening using IonOptix contractility system (IonOptix, Westwood, MA, USA). Cells were suspended in a specific chamber and placed on the microscope and then were field stimulated by constant 1 Hz depolarizing pulses from platinum electrodes placed on opposite sides of the cell chamber connected to a simulator. During experiments, cells were perfused with Krebs–Henseleit solution at a temperature of 37°C using a peristaltic pump. Cell shortening was assessed by percentage of cell shortening from 10 representative peaks from each phase of pharmacologic treatment.

### 2.5 FRET-based measurements of intracellular cAMP dynamics

The amount of cAMP produced following activation of different types of receptors was measured by a FRET-based method. Rat and human atrial cardiomyocytes were transduced with an adenovirus encoding the RII_Epac cAMP and pMEpac sensor and incubated for a minimum of 48 h prior to experiments.^24,25^ Atrial myocytes from CAG-Epac1-camps mice expressed the Epac1-cAMP biosensor endogenously, as mentioned above. Cav-3 overexpression and Cav3DN were delivered to the cell by 48 h pre-incubation at 37°C.^5^ Caveolae depletion was done by pre-treating the cells with methyl-β-cyclodextrin (MβCD, 13.1 mg/mL, Sigma-Aldrich, Gillingham, Dorset, UK), at 37°C for 45 min. YFP and CFP fluorescence were separated using a DualView splitter (Photometrics; D535/40, D430/30 filters) and captured by an ORCA-ER camera on a Nikon TE2000 inverted microscope. Images were acquired via Micro-Manager 1.4, and YFP/CFP ratios were calculated using a custom plug-in.^26^

### 2.6 Combined FRET/scanning ion conductance microscopy measurements of local cAMP signalling

Topographical imaging was performed using scanning ion conductance microscopy (SICM) with nanopipettes to visualize surface features such as T-tubules and crests. To isolate β_1_AR responses, β_2_ARs were blocked with ICI118,551 (50 nM), followed by bath-applied isoproterenol. Whole-cell FRET was continuously recorded via Micro-Manager. Local A_1_AR activation was then achieved by voltage-triggered SICM nanopipette delivery of 1 µM 2-MeCCPA ((2-chloro-N6-cyclopentyladenosine)) to either T-tubule or crest sites, guided by SICM topography.^27^

### 2.7 Super-resolution scanning patch-clamp with pipette clipping modification

For a single LTCC measurement, after generating a topographical image of the cell surface by SICM, the tip diameter of the pipette was widened by clipping to increase the area of attachment. The pipette was then lowered to a specific location until it touched the membrane, and a high resistance seal was established. Recordings were then performed in a cell-attached mode.^28^

### 2.8 Optical mapping of calcium transients

Calcium transients (CaTs) were recorded in (i) isolated mouse atrial preparations loaded with the calcium-sensitive dye Rhod-2 AM (10 μM, Invitrogen), immobilized with blebbistatin (10–20 μM, Tocris), electrically paced at 10 Hz, and imaged at 37°C using a MiCAM Ultima-L CMOS camera (SciMedia, Costa Mesa, CA, USA, 100 μm/pixel, 500–1000 fps) as previously described^29^ and (ii) isolated rat atrial myocytes loaded with Fluo-4 AM (10 μM, Invitrogen, Paisley, UK), electrically paced at 1 Hz and imaged on an inverted Nikon Eclipse Ti microscope equipped with a MiCAM Ultima-L camera (1 μm/pixel, 500–1000 fps).^14^

### 2.9 Quantitative real-time PCR

Total RNA was isolated from atrial tissue using the peqGOLD Total RNA Isolation Kit (Peqlab, Germany) per the manufacturer’s instructions. RNA purity and concentration were assessed via NanoDrop 2000 (Thermo Fisher, Paisley, UK). cDNA was synthesized from 1 µg RNA using the High-Capacity cDNA Reverse Transcription Kit (Applied Biosystems, Foster City, CA, USA). Quantitative reverse transcriptase–PCR was performed using SYBR® Green JumpStart™ Taq ReadyMix™ (Sigma-Aldrich, Gillingham, Dorset, UK) on a Mastercycler ep realplex system (Eppendorf, Hamburg, Germany). Thermal cycling conditions included 95°C for 10 min, followed by 40 cycles of 95°C for 15 s and 60°C for 60 s. Gene-specific primers are listed in Supplementary material online, *Table S1*. Relative gene expression was calculated using the 2^−ΔΔCt^ method, normalized to GAPDH. All reactions were run in triplicate and expressed as fold change relative to control.

### 2.10 Quantification and statistical analysis

All graphs and statistical analyses were performed using either GraphPad Prism 5 or Origin version 6.1. The average values were calculated throughout all cells studied within the groups and then compared between the groups. Normality was tested using the Kolmogorov–Smirnov test. In cases where data failed the normality test, the non-parametric Mann–Whitney *U* test was used instead of the unpaired Student’s *t*-test, and the non-parametric Kruskal–Wallis test was used instead of analysis of variance (ANOVA). Statistical differences were assessed with ANOVA, Student’s *t*-test, Mann–Whitney *U* test, Kruskal–Wallis test, χ^2^, and Fisher’s exact test as appropriate. All data are expressed as mean ± standard error of the mean (SEM). The value of *P* < 0.05 was considered statistically significant.

## 3. Results

### 3.1 A_1_AR is the dominant adenosine receptor subtype in atria and mediates inhibition of β_1_AR-driven cAMP signalling

**Figure 1:**
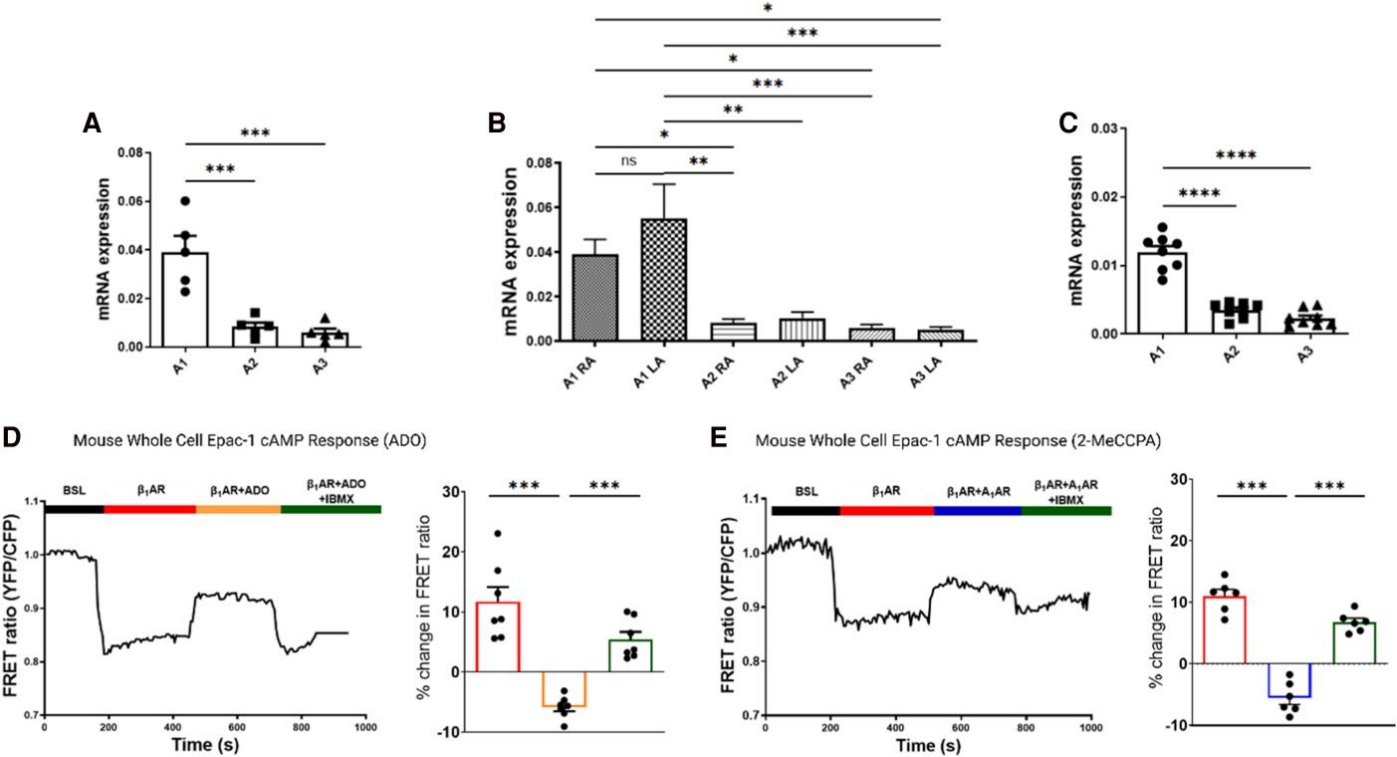
A₁ adenosine receptors predominate in atrial tissue and mediate inhibition of β₁AR-driven cAMP signalling. (*A*) qPCR quantification of adenosine receptor subtype mRNA expression (A₁AR, A₂AR, and A₃AR) in healthy rat atrial tissue (*N* = 5 rats). (*B*) Regional expression of A₁AR, A₂AR, and A₃AR in rat right atrium (RA) and left atrium (LA) (*N* = 5 rats). (C) Adenosine receptor subtype expression in donor human atrial tissue (*N* = 8 patients). Transcript abundance was normalised to the housekeeping gene and calculated using the ΔΔCt approach. (*D,E*) Whole-cell Epac1-camps FRET measurements in mouse atrial cardiomyocytes showing β₁AR-stimulated cAMP responses (50 nM ICI118,551 + 1 μM isoproterenol) followed by adenosine (20 μM; *D*) or selective A₁AR activation with 2′-MeCCPA (1 μM; *E*), and finally IBMX (100 μM); representative traces (left) and quantification of percentage change in FRET ratio (right) (*N* = 4 mice, *n* = 7 cells). Data are mean ± SEM; statistics by one-way ANOVA with Tukey's post hoc test; *P < 0.05, **P < 0.01, ***P < 0.001, ****P < 0.0001; ns, not significant.

Previous work has established the A_1_AR as a key mediator of adenosine’s anti-adrenergic effects in the heart, in both atrial and ventricular myocardium, acting via G_i_-coupled inhibition of adenylyl cyclase and suppression of cAMP signalling.^30^ However, the distribution of adenosine receptor subtypes within atrial tissue remains incompletely characterized, and it is unclear whether endogenous adenosine can directly restrain β_1_AR-driven cAMP signalling in atrial cardiomyocytes. To address this, we first quantified adenosine receptor subtype expression by qPCR in healthy rat atrial tissue and in donor human atrial tissue obtained from patients without clinical evidence of HF or atrial fibrillation. In rat atria, A_1_AR mRNA expression was significantly higher than A_2_AR and A_3_AR (*Figure 1A*), indicating that A_1_AR is the predominant adenosine receptor subtype. This expression hierarchy was maintained across both left atrium (LA) and right atrium (RA), with comparable A_1_AR levels between chambers and substantially lower expression of A_2_AR and A_3_AR (*Figure 1B*). A similar pattern was observed in human atrial tissue (*Figure 1C*), supporting conservation of dominant A_1_AR expression across species.

We next tested whether adenosine restrains β_1_AR-mediated cAMP production in atrial cardiomyocytes using FRET imaging in atrial myocytes isolated from Epac1-camps transgenic mice expressing a cytosolic cAMP sensor. β_1_AR stimulation with isoproterenol (1 μM) in the presence of the β_2_AR antagonist ICI118,551 (50 nM) produced a robust increase in FRET ratio (*Figure 1D* and *E*). Subsequent application of adenosine (20 μM) significantly reduced the β_1_AR-induced cAMP signal (*P* < 0.01; *Figure 1D*). Selective activation of A_1_AR with 2-MeCCPA (1 μM) elicited a comparable suppression of the β_1_AR response (*Figure 1E*), confirming that the anti-adrenergic effect was mediated via A_1_ARs, consistent with the expression data. In both protocols, addition of the non-selective phosphodiesterase inhibitor IBMX (3-isobutyl-1-methylxanthine) (100 μM) increased the signal to maximal levels (*P* < 0.001), serving as a saturating control. Together, these findings establish A_1_AR as the dominant adenosine receptor subtype in rat and human atrial tissue and demonstrate that endogenous adenosine and selective A_1_AR activation suppress β_1_AR-driven cAMP signalling in atrial cardiomyocytes.

### 3.2 A_1_AR stimulation suppresses β_1_AR-driven contraction by inhibiting cAMP signalling in sarcomeric microdomains in healthy rat atrial cardiomyocytes

**Figure 2:**
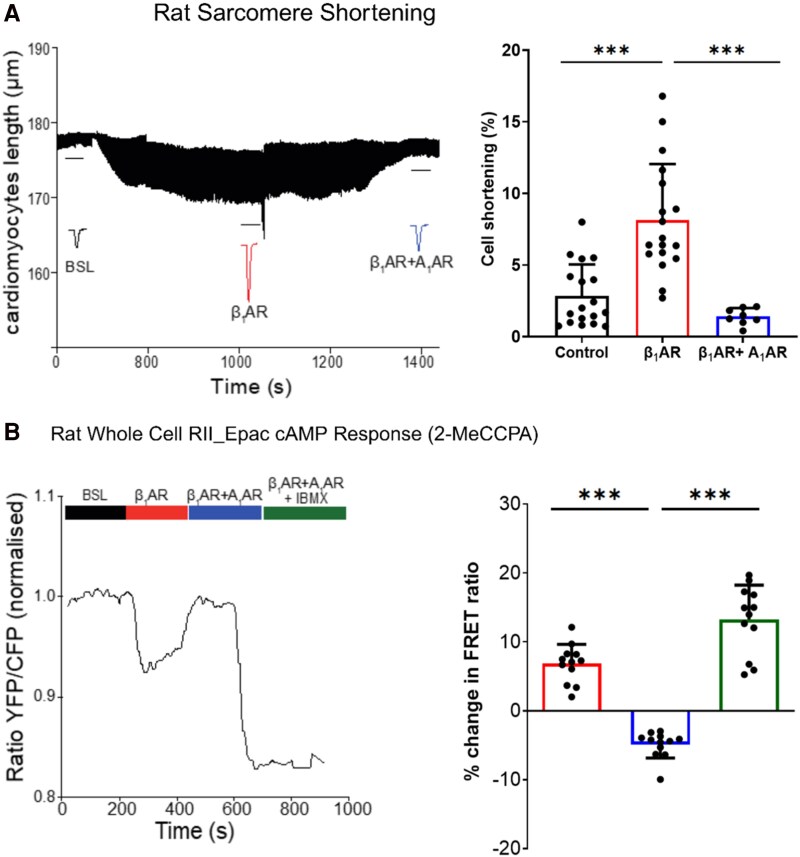
Suppression of β₁-adrenergic contraction and cAMP signalling by A₁AR stimulation in rat atrial cardiomyocytes. (*A*) Left: Representative cell shortening trace in a rat atrial cardiomyocyte showing increased contraction after β₁AR stimulation (50 nM ICI118,551 + 1 μM isoproterenol) and its suppression following A₁AR activation with 2-MeCCPA (1 μM). Right: Data showing cell shortening amplitude under each condition (*N* = 5 rats, *n* = 8 cells per group). (*B*) Left: Representative trace of cAMP FRET signal (RII_Epac sensor) from a rat atrial cardiomyocyte showing responses to β₁AR stimulation, A₁AR activation, and non-selective phosphodiesterase inhibition with IBMX (100 μM). Right: Quantification of FRET ratio changes for each condition (*N* = 5 rats, *n* = 10 cells per group). Data are presented as mean ± SEM. ***P < 0.001 by one-way ANOVA with Tukey's post hoc test.

To determine whether A_1_AR activation suppresses β_1_AR-driven contractile responses, we assessed sarcomere shortening and cAMP levels specifically within sarcomeric microdomains. Using IonOptix contractility test, β_1_AR stimulation (50 nM ICI 118,551 + 1 μM isoproterenol) significantly increased sarcomere shortening in field-stimulated atrial cardiomyocytes (*P* < 0.001; *Figure 2A*). Upon addition of the selective A_1_AR agonist 2-MeCCPA (1 μM), this β_1_AR-induced increase in sarcomere shortening was markedly reduced (*P* < 0.001 vs. β_1_AR), confirming a robust anti-adrenergic effect at the level of contractile function.

To assess local cAMP dynamics, we employed the RII_Epac FRET-based biosensor, which selectively detects cAMP within PKA Type II–enriched compartments localized near the sarcomere, regions critical for EC-coupling and myofilament regulation.^31^ The same pharmacological protocol was performed in FRET experiments, in which β_1_AR stimulation led to a significant increase in the RII_Epac FRET ratio from baseline (*P < 0.001*; *Figure 2B*). Activation of A_1_AR with 2-MeCCPA significantly reduced this β_1_AR-induced FRET signal (*P* < 0.001 vs. β_1_AR), indicating effective local suppression of cAMP. Subsequent application of IBMX (100 μM), a non-selective phosphodiesterase inhibitor, caused a substantial elevation in FRET ratio, consistent with maximal cAMP accumulation and serving as a normalization control. Together, these findings confirm that A_1_AR activation reduces β_1_AR-mediated increases in contractility and locally suppresses cAMP production in PKA Type II–enriched sarcomeric microdomains.

### 3.3 Local A_1_AR activation reduces β_1_AR-mediated cAMP signalling and LTCC activity at both crest and T-tubule microdomains

**Figure 3:**
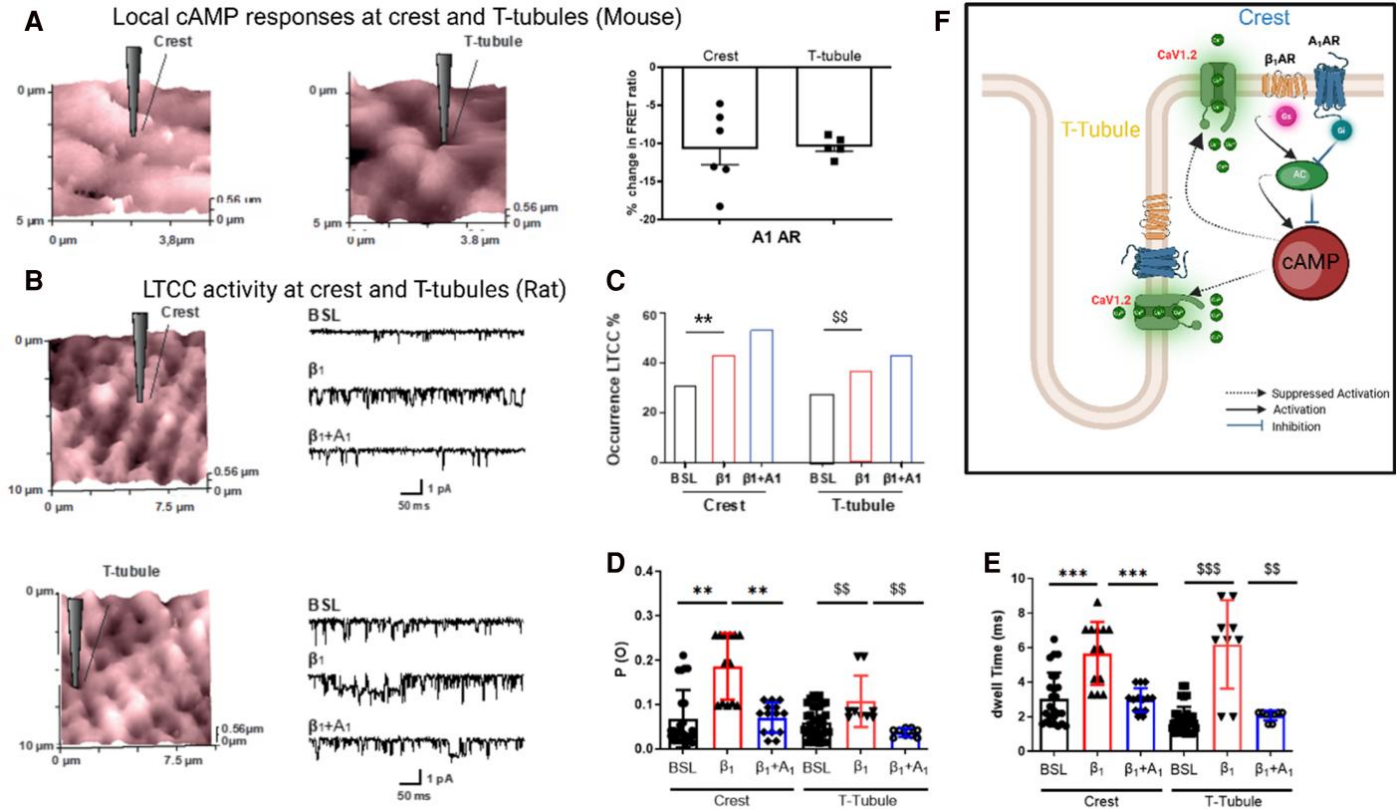
Localized A₁AR signalling suppresses β₁AR-mediated activity in both crest and T-tubule membrane domains of atrial myocytes. SICM-guided FRET imaging in atrial myocytes from Epac1-camps transgenic mice revealed that local stimulation of A₁ARs (1 μM 2′-MeCCPA) following global β₁AR activation (50 nM ICI118,551 + 1 μM isoproterenol) reduced whole-cell FRET signal similarly at crest and T-tubule regions (*A*; *N* = 4 mice, *n* = 8 cells per group). Scanning patch-clamp recordings of single LTCCs in healthy rat atrial myocytes revealed increased channel occurrence, Pₒ, and dwell time following β₁AR stimulation, with subsequent A₁AR activation reducing Pₒ and dwell time at both crest and T-tubule locations without affecting channel occurrence (*B-E*; *N* = 5 rats, *n* = 9-36 channels per group). (*F*) Schematic summarizing compartmentalized β₁AR-cAMP signalling at crest and T-tubule membranes and its suppression by A₁AR activation. **P < 0.01, ***P < 0.001 indicate significance at crest regions; $*P* < 0.001 indicates significance at T-tubule regions.

To determine whether A_1_AR-mediated inhibition of β_1_AR signalling is spatially restricted within the sarcolemma, we first combined SICM-guided topography with FRET-based cAMP imaging in atrial myocytes from Epac-cAMP transgenic mice.^27^ Local application of the A_1_AR agonist 2′-MeCCPA (1 μM) was performed at either crest or T-tubule regions following whole-cell β_1_AR stimulation (50 nM ICI118,551 + 1 μM isoproterenol). Both sites showed comparable reductions in the FRET signal, with no significant difference between membrane domains (*Figure 3A*), suggesting that A_1_ARs exert anti-adrenergic control at both locations.

To examine functional consequences at the channel level, we next recorded single-channel LTCC activity using SICM-guided cell-attached patch-clamp in isolated rat atrial myocytes. At baseline, LTCC open probability (*P*_o_) and occurrence were similar between crest and T-tubule recordings, as we previously reported for both rat and human atrial cardiomyocytes.^14^ β_1_AR stimulation markedly increased both LTCC occurrence and *P*_o_ at both sites (*P* < 0.01). Co-application of 2′-MeCCPA reduced LTCC *P*_o_ at both the crest (*P* < 0.01) and T-tubule (*P* < 0.05) membranes, without significantly affecting channel occurrence (*Figure 3B–D*). Consistent with this, β_1_AR stimulation prolonged mean channel open time (dwell time), and A_1_AR co-stimulation reversed this prolongation at both crest and T-tubule sites (*Figure 3E*). These findings indicate that A_1_ARs are functionally active in both sarcolemma microdomains and suppress β_1_AR-driven LTCC activity in a spatially uniform manner.

### 3.4 Impaired A_1_AR-mediated anti-adrenergic response in HF

**Figure 4:**
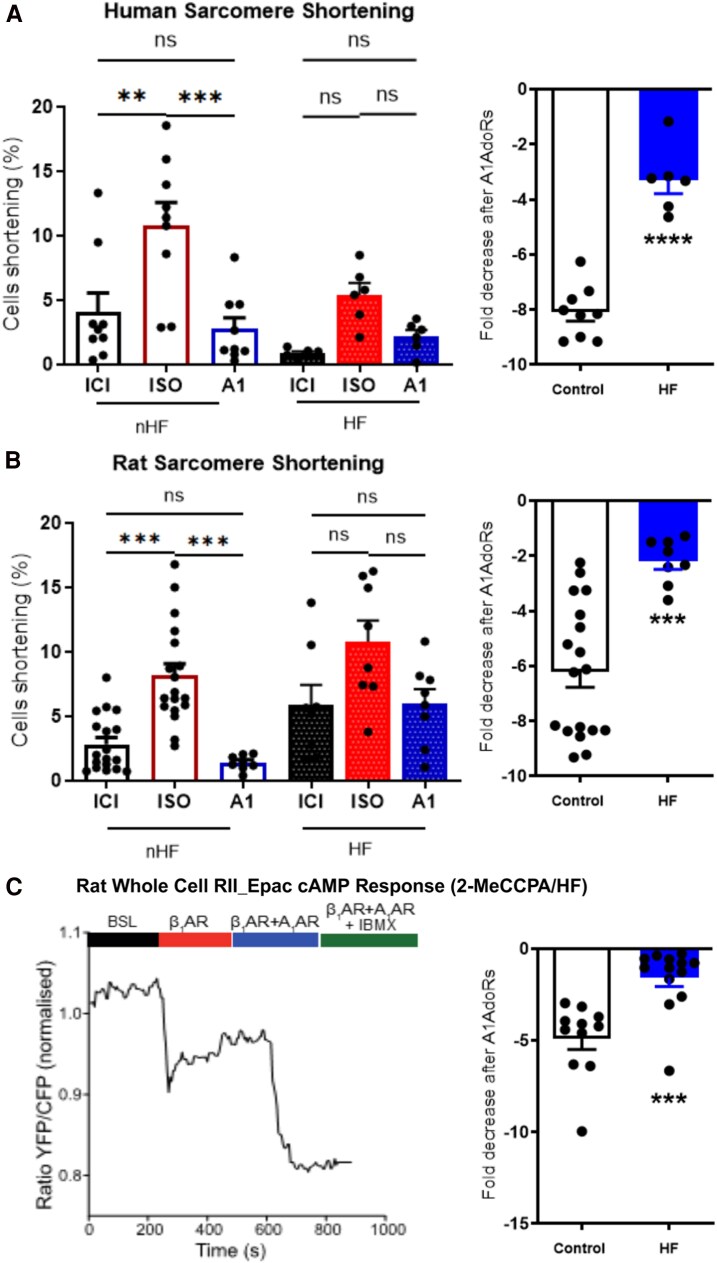
Impaired anti-adrenergic response to A₁AR stimulation in atrial myocytes from failing human and rat hearts. (*A*) Sarcomere shortening in human atrial myocytes from non-failing (nHF) and heart failure (HF) patients following β₁AR stimulation and subsequent A₁AR activation. Right: Fold decrease in contraction amplitude in response to A₁AR stimulation (*N* = 5 patients, *n* = 6-9 cells per group). (*B*) Sarcomere shortening responses under identical stimulation protocols in atrial myocytes from control and 16 weeks post-MI HF rats (*N* = 5 rats, *n* = 8-18 cells per group). (*C*) FRET-based cAMP measurements using the RII-Epac sensor in isolated rat atrial myocytes. Left: Representative trace showing sequential β₁AR stimulation, A₁AR activation, and maximal response following phosphodiesterase inhibition. Right: Fold decrease in FRET signals following A₁AR stimulation (*N* = 5 rats, *n* = 11-13 cells per group). Data are mean ± SEM. ***P < 0.001, ****P < 0.0001 by one-way ANOVA with Tukey's post hoc test or unpaired t-test, as appropriate.

**Figure 5:**
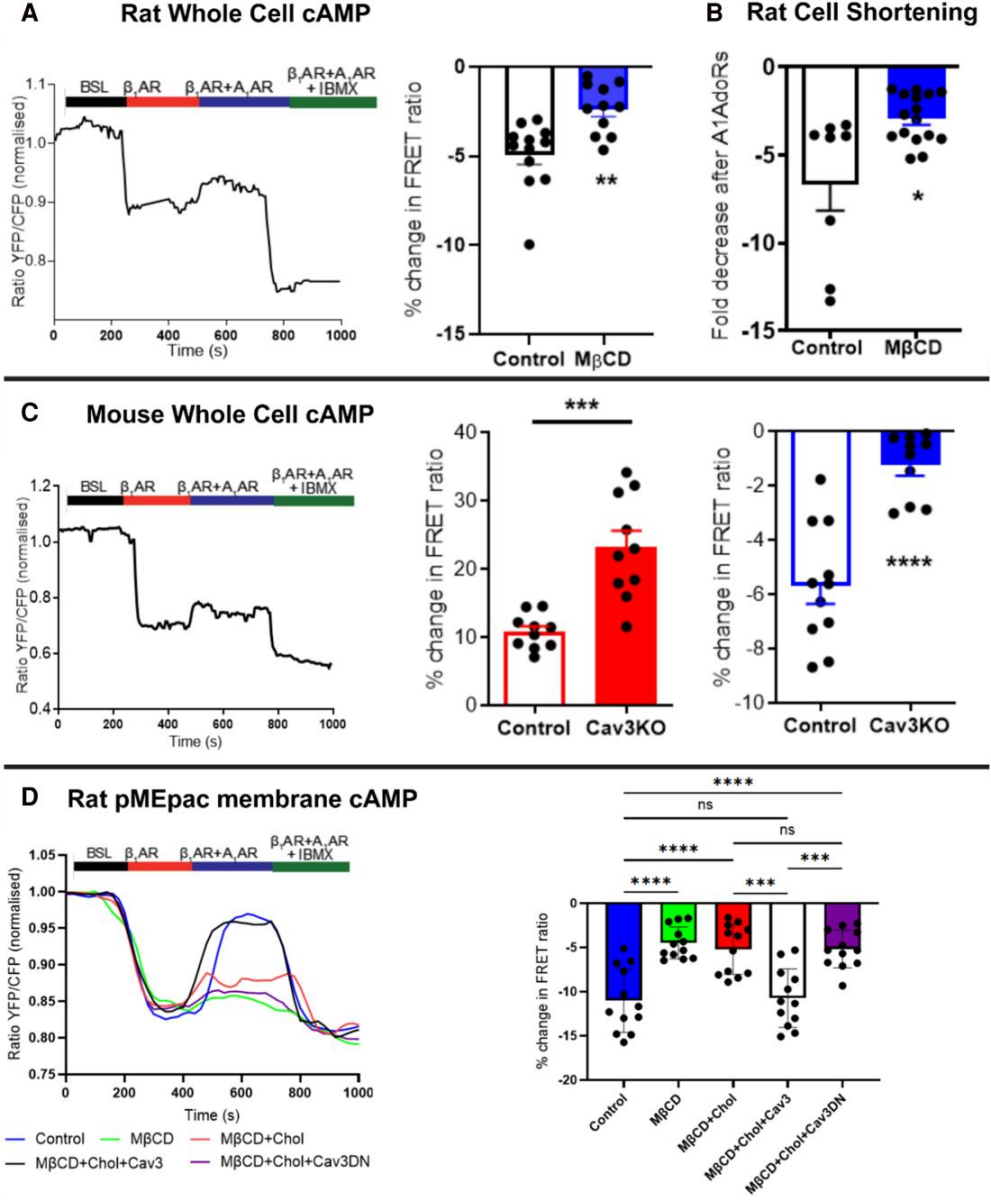
Caveolar disruption impairs A₁AR-mediated anti-adrenergic signalling in atrial myocytes. (*A*) Whole-cell Epac-based FRET recordings in rat atrial myocytes showing β₁AR-stimulated cAMP responses (50 nM ICI118,551 + 1 μM isoproterenol) followed by A₁AR activation (2′-MeCCPA, 1 μM) and IBMX (100 μM); quantification shows reduced A₁AR-mediated cAMP suppression after MβCD treatment. (*B*) Sarcomere shortening measurements in the same experimental groups showing attenuated A₁AR-dependent reduction in β₁AR-stimulated contraction following MβCD. (*C*) Whole-cell cAMP imaging in atrial myocytes from Cav3KO mice showing enhanced β₁AR-driven cAMP responses but loss of A₁AR-mediated suppression; representative trace (left) and quantification (middle/right). (*D*) Membrane-targeted pMEpac FRET recordings in rat atrial myocytes showing that MβCD blunts A₁AR-mediated suppression of β₁AR-driven membrane cAMP; cholesterol repletion alone does not restore this response, whereas Cav3 re-expression restores A₁AR-dependent cAMP suppression, and Cav3DN fails to do so; representative traces (left) and quantification (right). *P*-values were determined by unpaired t-tests for two-group comparisons (*A* and *B*), unpaired t-tests for Cav3KO comparisons (*C*), and one-way ANOVA with post hoc multiple comparisons for pMEpac conditions (*D*). Sample sizes: rat (*A* and *B*) *N* = 5, *n* = 11-12 (cAMP) and *n* = 8-15 (shortening); mouse (*C*) *N* = 4, *n* = 10; rat pMEpac (*D*) *N* = 5, *n* = 12. *P < 0.05, **P < 0.01, ***P < 0.001, ****P < 0.0001; ns, not significant.

To determine whether impaired A_1_AR responsiveness in HF reflects altered receptor or caveolar expression, we first quantified A_1_AR and Cav3 mRNA levels in rat and human atrial tissue. In rats, neither A_1_AR nor Cav3 transcript abundance differed between control and HF groups (see Supplementary material online, *Figure S1*), indicating that loss of A_1_AR function in HF is unlikely to arise from reduced gene expression. We therefore next assessed whether A_1_AR-mediated anti-adrenergic signalling is functionally preserved by comparing contractile and cAMP responses in atrial cardiomyocytes isolated from non-failing donors (nHF) and HF human patients and from the same rat HF model. In human atrial cardiomyocytes, β_1_AR stimulation significantly enhanced sarcomere shortening in the nHF donor group while this stimulatory effect was significantly diminished in failing cells (*Figure 5A*, left panel). Following A_1_AR activation, contractility was reduced in nHF but not in HF groups (nHF: *P* < 0.01; HF: *P*  *=*  *0.592*). The fold decrease in sarcomere shortening after A_1_AR stimulation was significantly lower in HF compared with nHF (*P* < 0.05; *Figure 4A*, right panel). A comparable pattern was observed in atrial cardiomyocytes isolated from control (non-failing) and HF rats (*Figure 4B*). β_1_AR stimulation enhanced sarcomere shortening in both groups, followed by a reduction after A_1_AR stimulation. However, the extent of suppression was significantly attenuated in HF myocytes (*P* < 0.05; *Figure 4B*, right panel). While non-failing myocytes exhibited a clear decline in contraction after A_1_AR activation, HF myocytes showed only a modest and statistically non-significant reduction (β_1_AR vs. A_1_AR, *P* = 0.058), confirming impaired A_1_AR responsiveness in failing myocardium.

To investigate the signalling mechanism underlying this functional attenuation, we measured cytosolic cAMP using the RII-Epac FRET biosensor in isolated rat atrial cardiomyocytes (*Figure 4C*). A_1_AR activation significantly suppressed β_1_AR-driven cAMP levels in both groups, but the magnitude of suppression was significantly reduced in HF myocytes (*P*  *<* 0.001). The subsequent application of IBMX produced similar maximal FRET signals in both groups, confirming preserved overall cAMP production capacity. Together, these findings demonstrate that A_1_AR-mediated anti-adrenergic suppression of both contractility and cAMP production is significantly diminished in atrial cardiomyocytes from both human patients and a rat HF model.

### 3.5 Caveolar disruption attenuates A_1_AR-mediated suppression of β_1_AR–cAMP signalling and contractility

Previous studies have shown that A_1_ARs localize to caveolar membrane compartments.^32^  *In vitro*, the A_1_AR C-terminal domain directly binds Cav-1, a key caveolar scaffolding protein.^12^ These findings support a model in which caveolae serve as specialized signalling platforms that coordinate A_1_AR-mediated regulation of cardiac excitability and function. To assess whether caveolar microdomains contribute to A_1_AR-dependent anti-adrenergic signalling, we disrupted caveolae in adult rat atrial myocytes using 10 mM MβCD for 30 min at room temperature.^14,33^ Following β_1_AR stimulation, A_1_AR activation induced a significantly smaller reduction in cAMP levels in MβCD-treated cells compared to controls (*P* < 0.05; *Figure 5A*). A parallel reduction in contractile response was observed, with A_1_AR stimulation producing a markedly blunted decrease in sarcomere shortening in MβCD-treated cells (*Figure 5B*), supporting a functional role for membrane caveolae in local anti-adrenergic signalling.

To further probe the structural role of caveolae in this signalling, we employed double transgenic CAG-Epac1-camps/Cav3KO mice, in which cardiac-specific conditional deletion of Cav3 abolishes caveolar formation while allowing endogenous expression of the Epac1-cAMP biosensor for live-cell cAMP imaging. Atrial cardiomyocytes from these mice exhibited a significantly greater cAMP response to β_1_AR stimulation (*P* < 0.05), yet A_1_AR activation failed to reduce this signal (*P* < 0.01), confirming the loss of A_1_AR-dependent inhibition in the absence of functional caveolae (*Figure 5C*).

Finally, to probe whether restoring Cav3-dependent organization can recover A_1_AR function, we measured membrane-local cAMP using pMEpac in rat atrial myocytes. MβCD markedly reduced the A_1_AR-dependent suppression of β_1_AR-driven membrane cAMP (*P* < 0.001), and cholesterol repletion alone did not restore the response (*Figure 5D*, *P* = 0.967). In contrast, Cav3 overexpression in cholesterol-repleted cells reinstated A_1_AR-mediated cAMP suppression (*P*  *<* 0.001), whereas expression of Cav3DN did not (*Figure 5D*, *P* = 0.974). Collectively, these data indicate that Cav3-dependent caveolar organization is required for effective A_1_AR anti-adrenergic control of β_1_AR–cAMP signalling and contractile responses in atrial myocytes.

**Figure 6:**
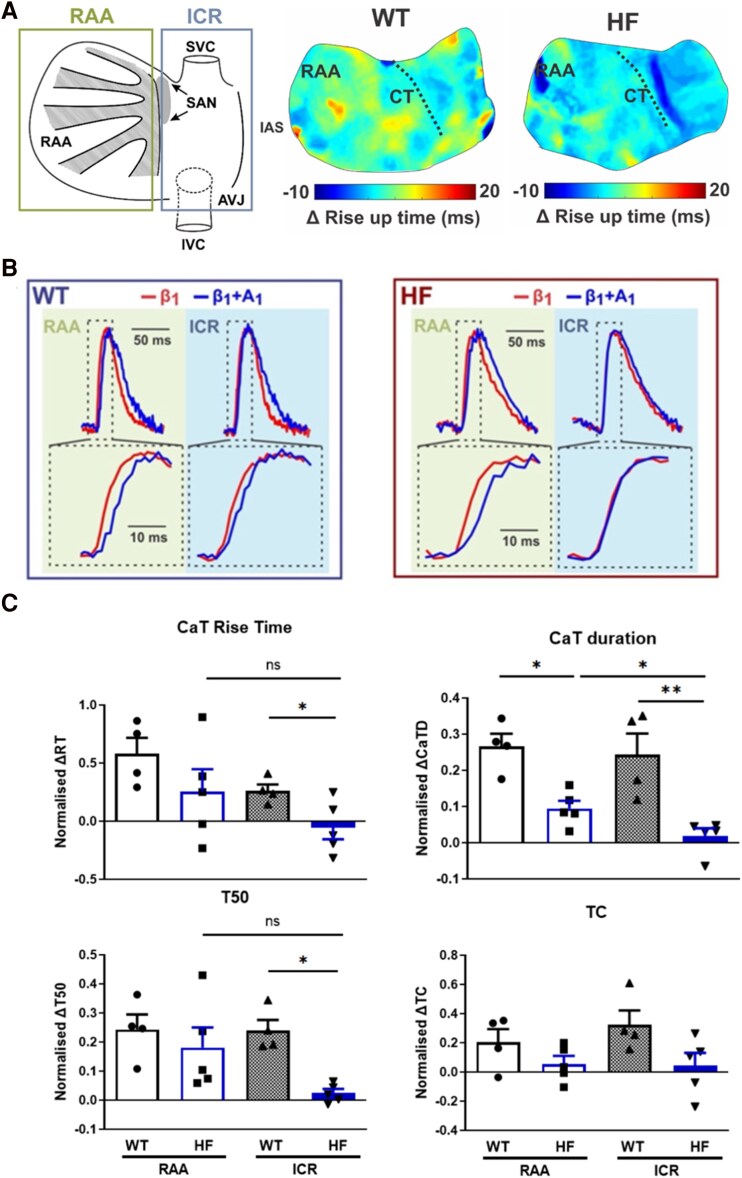
Regional variation in A₁AR-mediated anti-adrenergic regulation of CaTs in control and HF atria. (*A*) Left: Schematic of the isolated mouse atrial preparation used for CaT recordings. The right atrial appendage (RAA) and intercaval region (ICR) are delineated for regional analysis. Right: Representative CaT rise-up time map from control and HF atria, showing regional heterogeneity in Ca²⁺ cycling. (*B*) Representative CaT traces from the RAA and ICR in WT (left) and HF (right) mice. Enlarged CaT upstrokes (bottom panels) illustrate changes in CaT rise-up time. (*C*) Summary of A₁AR-induced changes in CaT characteristics, presented as relative changes normalised to the preceding β₁AR stimulation. Top left: Normalized changes in CaT rise-up time. Bottom left: Normalized changes in time to 50% CaT decay (T50). Top right: Normalized changes in CaT duration (CaTD). Bottom right: Normalized changes in time constant (TC) of CaT decay. Data are mean ± SEM. *P < 0.05, **P < 0.01, ***P < 0.001 by one-way ANOVA with Tukey's post hoc test.

### 3.6 Regional anti-adrenergic effects of A_1_AR on calcium handling are selectively impaired in HF

The atria display significant regional heterogeneity in both structural organization and Ca^2+^ handling properties, which may influence receptor-mediated signalling. Recent studies have shown that the T-tubules are present in the RAA but largely absent in the ICR, in both mouse and human atria.^10^ This anatomical difference is expected to modulate the local coupling between LTCCs and ryanodine receptor type 2 (RyR2s), potentially creating region-specific regulation of adrenergic and A_1_AR-mediated anti-adrenergic signalling.

To investigate whether A_1_AR-mediated anti-adrenergic effects vary across atrial regions, we performed high-resolution fluorescent optical mapping of CaTs in mouse atrial preparations isolated from control (non-failing) and HF mice. The RA was divided into the RAA and ICR as defined in *Figure 6A*.

**Figure 7:**
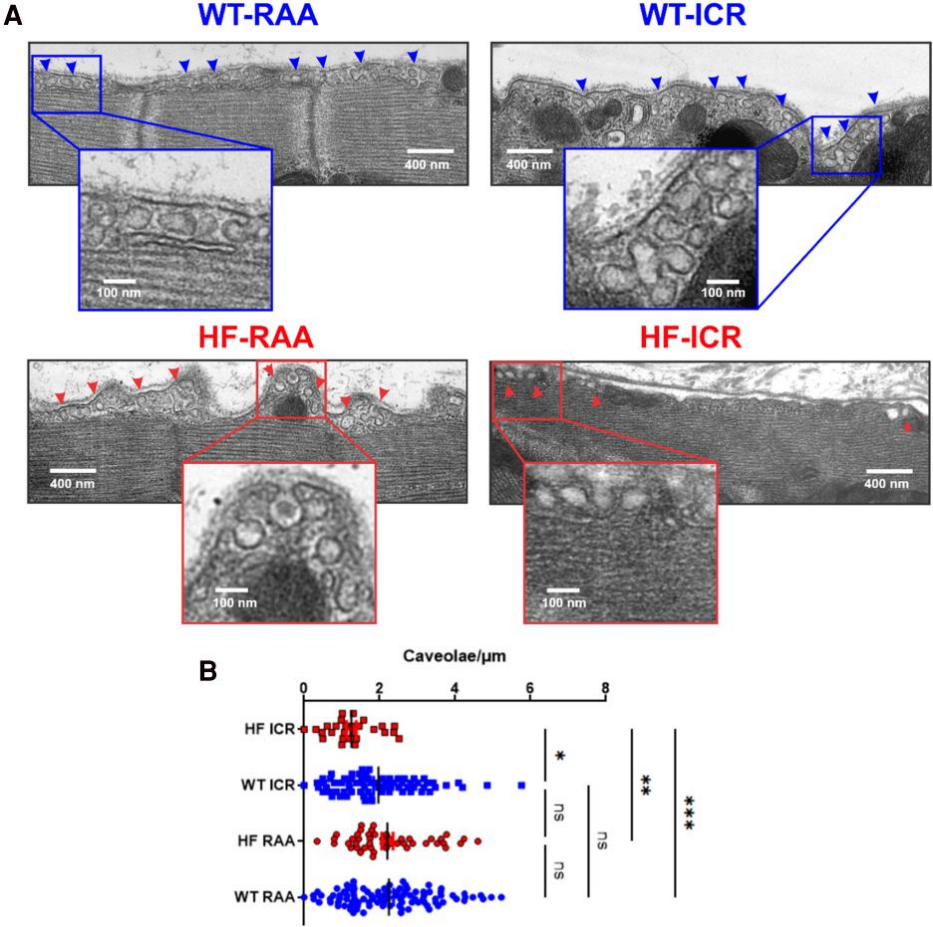
HF is associated with reduced caveolae density in the ICR. (*A*) Representative transmission electron micrographs of subsarcolemmal caveolae (arrowheads) in the right atrial appendage (RAA) and intercaval region (ICR) from WT and HF hearts; boxed regions are shown enlarged. Scale bars, 400 nm (main images) and 100 nm (insets). (*B*) Caveolae density (caveolae/μm sarcolemma) quantified in WT-RAA, HF-RAA, WT-ICR, and HF-ICR; *N* = 3-5 mice, *n* = 20-30 images per heart. *P < 0.05, **P < 0.01, ***P < 0.001 by one-way ANOVA with Tukey's post hoc test.

Quantification of the normalized change in CaT kinetics following addition of A_1_AR stimulation (relative to β_1_AR alone) is summarized in *Figure 6C*. In wild-type (WT) atria, A_1_AR activation produced clear slowing of CaT activation in both regions, reflected by positive shifts in rise time and T50 in the RAA and ICR. In HF, this A_1_AR-dependent slowing was preserved in the RAA (rise time: *P*  *=* 0.231, T50: *P* = 0.518), but was significantly reduced in the ICR, where rise time (*P* = 0.040) and T50 (*P* = 0.026) responses were largely abolished. A_1_AR stimulation also prolonged CaT duration (CaTD) in WT preparations, whereas this effect was attenuated in HF in both regions and was minimal in the HF-ICR (HF-RAA: *P* = 0.024; HF-ICR: *P* = 0.003). In contrast, time constant (TC) of CaT decay was not detectably altered across groups. Together, these data indicate a selective loss of A_1_AR-mediated anti-adrenergic control in the ICR in HF. Because the ICR is relatively T-tubule-poor, signalling control of Ca^2+^ handling is expected to depend more heavily on peripheral sarcolemmal microdomains, including caveolae, which we found to be required for A_1_AR anti-adrenergic signalling in isolated myocytes.

### 3.7 HF is associated with reduced caveolae density in the ICR

Based on this functional dependence on caveolar integrity, we next asked whether caveolar ultrastructure differs between atrial regions and whether it is preferentially disrupted in HF. Transmission electron microscopy was performed in the RAA and ICR tissues from WT and HF hearts to visualize subsarcolemmal caveolae. Representative micrographs show characteristic flask-shaped caveolar invaginations along the sarcolemma in both WT-RAA and WT-ICR (*Figure 7A*). Quantification of caveolae density indicated no significant regional difference in WT atria (WT-RAA vs. WT-ICR; *Figure 7B*). In HF, however, caveolae remain in the RAA, but were markedly reduced in the ICR, where long stretches of sarcolemma lacked discernible caveolar profiles (*Figure 7B*).

Quantification of caveolae density confirmed significant regional and disease-dependent differences (*Figure 8B*). Together, these data demonstrate a selective loss of caveolae in the T-tubule-poor ICR in HF, consistent with the notion that structural disruption of caveolar microdomains contributes to the regional impairment of A_1_AR anti-adrenergic signalling observed in failing atria.

## 4. Discussion

HF is characterized by chronic sympathetic overactivation and maladaptive remodelling of intracellular signalling pathways, particularly within the atria, where Ca^2+^ signalling dysregulation contributes to both impaired contractile function and heightened arrhythmogenic risk. Concomitantly, myocardial adenosine levels are known to increase in HF due to enhanced Adenosine triphosphate breakdown and reduced clearance, suggesting a compensatory role for adenosine signalling in restraining adrenergic overstimulation. A_1_ARs, which inhibit β_1_-adrenergic receptor (β_1_AR) driven cAMP production via G_i_-coupled mechanisms, are thought to mediate this protective effect. However, the spatial organization of A_1_AR signalling within atrial cardiomyocytes and its susceptibility to disruption in the failing heart has remained poorly defined. In this study, we show that A_1_AR-mediated inhibition of β_1_AR signalling is not uniformly distributed but instead most effective within Cav3-dependent caveolar signalling domains. This compartmentalized regulation is disrupted in HF, particularly in atrial regions deficient in T-tubules. These findings establish a multiscale framework from nanodomain to regional tissue level in which structural remodelling underlies the loss of A_1_AR anti-adrenergic control in HF.

### 4.1 A_1_AR–β_1_AR functional coupling is spatially restricted

Using Epac-based FRET biosensors targeted to the cytosol (Epac1-camps) and PKA Type II–anchored compartments (RII_Epac), we show that β_1_AR stimulation increases cAMP levels broadly across both compartments (*Figures 1D* and *E* and *3B*). However, A_1_AR activation significantly suppresses this response, with a greater effect detected by the membrane-associated RII_Epac sensor. These data indicate that, although β_1_AR-driven cAMP is generated broadly, A_1_AR–G_i_ signalling acts most effectively within PKA Type II–anchored nanodomains that are aligned with the EC axis. In this context, the RII_Epac sensor reports a functionally local cAMP pool defined by PKA anchoring and associated signalling complexes rather than a strictly anatomical compartment, consistent with the concept that G protein-coupled receptor (GPCR) signalling in cardiomyocytes is organized into nanoscopic ‘signalosomes’.^20,34^ Collectively, our data extend the physiological role of A_1_ARs beyond nodal conduction and heart rate control^35–37^ to include spatially confined regulation of β_1_AR-driven signalling relevant to atrial EC-coupling.

### 4.2 Caveolar microdomains are essential for A_1_AR-mediated anti-adrenergic signalling

Our findings demonstrate that caveolae are essential for A_1_AR-mediated inhibition of β_1_AR-induced cAMP accumulation, LTCC *P*_o_ and contractility (*Figures 2–4*). Disruption of caveolae using MβCD or genetic deletion of Cav3 abolished A_1_AR signalling effects (*Figure 5*), consistent with prior work identifying Cav3 as a central scaffold for GPCR compartmentation and β_2_AR–G_i_ crosstalk.^5,12,32,38^ In support of a structural requirement for Cav3-dependent organization, overexpression of Cav3 partially restored A_1_AR-mediated suppression of β_1_AR-stimulated cAMP signalling in caveolae-disrupted atrial myocytes, whereas cholesterol repletion alone or expression of dominant-negative Cav3 did not (*Figure 5D*).

Co-immunoprecipitation studies have previously revealed physical associations between A_1_ARs and Cav3,^32,39^ supporting the hypothesis that A_1_ARs are localized within caveolar complexes and rely on these structures for G_i_ coupling and spatial restriction of adenylyl cyclase activity. Together, these observations support a model in which Cav3-dependent caveolar organization provides a structural framework for efficient, localized A_1_AR anti-adrenergic signalling in atrial cardiomyocytes.

### 4.3 HF disrupts A_1_AR-mediated anti-adrenergic control

Our findings confirm that A_1_AR remains the predominant adenosine receptor subtype in both healthy and failing rat and human atrial tissues, in agreement with previous transcriptomic and proteomic studies of cardiac adenosine receptor distribution.^40^ Despite preserved expression (see Supplementary material online, *Figure S1*), A_1_AR-mediated suppression of β_1_AR-driven responses was markedly diminished in atrial myocytes isolated from both HF patients and rat models of HF. This was evident at both functional and signalling levels: A_1_AR activation failed to attenuate β_1_AR-stimulated contractility or cytosolic cAMP production, as assessed by sarcomere shortening assays and Epac-FRET imaging, respectively (*Figure*  *4*). These data are consistent with impaired receptor–effector coupling in HF, rather than simple loss of receptor abundance.

Several pathophysiological mechanisms may underlie this uncoupling. Chronic HF is associated with altered membrane lipid composition, including cholesterol depletion, redistribution of sphingolipids, and impaired Cav3 trafficking, all of which may destabilize caveolar structure and perturb GPCR nanodomain organization.^41,42^ Indeed, similar compartmental dislocation has been documented for β_2_ARs in failing myocardium, where loss of caveolar residency leads to impaired receptor function and aberrant downstream signalling.^43^ Although Ca_V_1.2 regulation is generally attributed to cAMP/PKA-dependent phosphorylation, Gβγ interactions with the Ca_V_1.2 α1 subunit have been reported in heterologous systems, suggesting a potential cAMP-independent route for G_i_ signalling to influence LTCC gating.^44^ Because G_i_ signalling components are enriched within caveolar membranes through caveolin-associated scaffolding,^45^ the loss/disruption of caveolae observed in our HF models could weaken local G_i_/Gβγ regulation of LTCCs in parallel with impaired compartmentalized cAMP control. Defining the relative contribution of these mechanisms will require dedicated experiments. Distinguishing between these pathways will be an important focus of future work. In this context, our findings support the interpretation that A_1_ARs remain present but become less effective at engaging inhibitory signalling within the relevant microdomains in HF.

### 4.4 Region-specific loss of A_1_AR regulation in HF linked to structural remodelling

Our findings show that disruption of A_1_AR signalling in HF is regionally determined by atrial structural organization. Optical mapping revealed that A_1_AR-mediated anti-adrenergic regulation of Ca^2+^ handling is preserved in the RAA but selectively lost in the ICR of failing hearts (*Figure 6*). This pattern mirrors membrane architecture: the RAA contains a more developed T-tubule network, whereas the ICR is comparatively T-tubule-poor, increasing reliance on peripheral sarcolemmal platforms such as caveolae and thereby vulnerability to HF-associated microdomain disorganization.^46^ Importantly, caveolae/Cav3 are not restricted to surface crests; Cav3-dependent signalling microdomains are also present at the T-tubular membrane, where they help organize local cAMP/PKA control of LTCC function.^47^

Consistent with a structural coupling between these systems, genetic loss of Cav3 disrupts T-tubule organization and alters the distribution of tubular LTCC current, supporting the idea that caveolar scaffolding contributes to T-tubule integrity.^48^ Consistent with this idea, TEM analysis (*Figure 7*) showed a marked reduction in subsarcolemmal caveolae density in HF-ICR, whereas caveolae were comparatively preserved in the RAA, providing ultrastructural support for a regionally selective loss of caveolar signalling capacity in HF.

Caveolae are well recognized as mechanosensitive membrane reservoirs that flatten under increased membrane tension and can undergo disassembly when membrane architecture or lipid organization is perturbed,^7,49–51^ which would be expected to weaken local GPCR signal integration in structurally simplified myocardium. It is also worth noting that A_1_AR association with caveolae appears to be dynamic rather than constitutive. In rat ventricular myocytes, biochemical fractionation showed that a substantial proportion of A_1_ARs reside in caveolar fractions at baseline, but agonist stimulation triggered rapid redistribution of A_1_ARs into non-caveolar membrane fractions, an effect prevented by A_1_AR antagonism.^52^ This activity-dependent trafficking has been discussed more broadly as part of a caveolae/caveolin role in organizing GPCR signalling and receptor routing at the sarcolemma. In the context of the present work, these observations support the view that caveolae can act as regulatory staging sites that constrain where and how A_1_AR–G_i_ signalling engages adenylyl cyclase, rather than simply marking a fixed receptor location.

At the cellular level, we further confirmed this structural–functional link using single-cell analysis of rat atrial myocytes classified by T-tubule density (see Supplementary material online, *Figure S2*). These data do not imply that A_1_AR signalling is exclusively T-tubule-dependent, since modulation was detected at both crest and T-tubule membrane sites in healthy myocytes; rather, T-tubule loss likely marks a broader structural simplification that increases dependence on caveolae-organized nanodomains for coordinating cAMP regulation and Ca^2+^ handling.

Clinically, RA triggers often arise from peri-caval/crista terminalis regions^53^ and the posterior atrium/ICR are recognized substrates for ectopy and atrial fibrillation (AF) initiation in structural heart disease and HF.^54^ Our recent work supports the view that the peri-caval region is particularly vulnerable in disease: in Cav3-deficient and post-MI HF mice, atrial ectopy increases and the leading pacemaker site shifts away from the sinoatrial node towards ectopic foci localized within the peri-caval region.^23^ This area is characterized by sparse T-tubules, disrupted conduction properties, and slower CaTs,^10^ as well as disease-mediated disruption of caveolae structures, and local loss of A_1_AR anti-adrenergic restraint may therefore contribute to regional Ca^2+^ dysregulation and arrhythmogenic vulnerability.

### 4.5 Electrophysiological implications of microdomain A_1_AR signalling

Adenosine provides an endogenous constraint on atrial EC-coupling during sympathetic drive by activating A_1_AR–G_i_ signalling to inhibit adenylyl cyclase, reduce cAMP production and downstream PKA activity, and oppose β_1_AR-dependent phosphorylation of EC-coupling targets. In this setting, the dominant consequence for contractile function is reduced cAMP/PKA-driven enhancement of Ca_V_1.2 and sarcoplasmic reticulum Ca^2+^ cycling, which lowers Ca^2+^ availability and contractile amplitude, while parallel effects on membrane currents can additionally reshape action potential configuration. In keeping with an anti-adrenergic effect on Ca_V_1.2, adenosine attenuates the isoprenaline-enhanced LTCC current in human atrial myocytes.^55^ In our SICM-guided single-channel recordings, A_1_AR stimulation reduced LTCC *P*_o_ after β_1_AR activation at both crest and T-tubule membranes (*Figure 3*), which fits well with reversal of PKA-dependent channel facilitation rather than a change in channel availability. More broadly, βAR/cAMP/PKA signalling is a central route for enhancing Ca_V_1.2 activity and shaping EC-coupling, providing a mechanistic bridge from the RII_Epac microdomain signals to downstream electrical and Ca^2+^ handling consequences.^56^

In parallel, A_1_ARs can activate G protein-gated inwardly rectifying potassium channel (GIRK) channels via Gβγ to generate *I*_K, Ado_, hyperpolarizing the membrane resting potential and abbreviating repolarization.^57^ In human atria, adenosine produces pronounced RA–LA asymmetry in action potential shortening and thus can promote sustained atrial fibrillation maintained by localized re-entrant drivers in RA regions with higher A_1_AR and GIRK4 expression; importantly, GIRK blockade (tertiapin) counteracts adenosine-induced action potential shortening and prevents atrial fibrillation induction in that setting. Recent electrophysiological and modelling work in pacemaker cells further supports a combined mechanism in which Gβγ-driven *I*_K,Ado_ and Giα-mediated suppression of cAMP/PKA-dependent Ca^2+^ cycling act synergistically to alter electrical behaviour; in this study, the combined effects on *I*_Ca,L_, *I*_f_, and *I*_K,Ado_ and sarcoplasmic reticulum Ca^2+^ handling produced substantially larger rate slowing than any single mechanism alone.^58^ Together, these pathways provide a direct link between microdomain-specific A_1_AR control of cAMP and changes in action potential waveform and arrhythmia vulnerability, particularly in HF where loss of local anti-adrenergic restraint may unmask pro-arrhythmic substrate.

Translational perspective Adenosine acting via A_1_ receptors provides an endogenous constraint on β-adrenergic signalling in the atria. We show that this protection relies on Cav3-dependent caveolar organization and is lost in HF, particularly in the ICR of the RA where caveolae density is downregulated. Diminished A_1_ anti-adrenergic control permits enhanced β_1_AR–cAMP–Ca^2+^ signalling and regional Ca^2+^ dysregulation, a substrate linked to atrial ectopy and atrial fibrillation in structural heart disease. These findings identify membrane microdomain integrity as a determinant of atrial autonomic balance and suggest that stabilizing caveolar organization may help restore adenosine-mediated restraint in HF.

## 5. Limitation and future work

A limitation of the human heart study is the uneven depth of clinical annotation. For each sample, we had access to basic demographics, operative indication, selected cardiovascular history, and pre-operative medications. However, more granular descriptors of HF severity such as left ventricular ejection fraction (LVEF) and B-type natriuretic peptide (BNP) values and arrhythmia burden were not available, so we could not test whether the degree of A_1_AR dysfunction varies with disease severity or treatment. In addition, although our experiments were designed to interrogate the anti-adrenergic, cAMP-dependent arm of A_1_AR signalling, A_1_AR activation has also been linked to other downstream pathways such as mitogen-activated protein kinase/extracellular signal-regulated kinase (MAPK/ERK); whether these outputs are similarly compartmentalized and remodelled in HF was not assessed here. The extent to which these non-cAMP pathways are compartmentalized within atrial microdomains, and whether they are remodelled in HF alongside loss of caveolar organization, remains to be determined.

## 6. Conclusion

A_1_ARs exert spatially organized anti-adrenergic control in atrial myocytes by restraining β_1_-adrenergic cAMP signalling within discrete membrane compartments. Using compartment-targeted biosensors and regional analysis, we show that this regulation is supported by Cav3-dependent caveolar organization and is modulated by local cellular architecture, including T-tubule heterogeneity. In HF, A_1_AR anti-adrenergic signalling is selectively lost in structurally vulnerable atrial regions such as the intercaval myocardium, consistent with microdomain disorganization rather than reduced receptor expression. This regional uncoupling is associated with impaired Ca^2+^ regulation and provides a mechanistic framework linking atrial structural remodelling to autonomic imbalance and arrhythmia susceptibility in disease. Preserving or restoring membrane microdomain organization may therefore represent a strategy to re-establish protective anti-adrenergic signalling and limit atrial dysfunction in HF.

## Supplementary Material

cvag131_Supplementary_Data

## Data Availability

The data underlying this article will be shared on reasonable request to the corresponding author.
