# Supplementary material for: Loss of caveolar A1 adenosine receptor signalling blunts anti-adrenergic control in heart failure
Source: Cardiovasc Res. 2026 Jun 24;122(10):1329–43. doi: 10.1093/cvr/cvag131 (PMC13355833; doi:10.1093/cvr/cvag131)
Supplement: cvag131_Supplementary_Data [file cvag131_supplementary_data.docx]

**EXPERIMENTAL MODEL AND SUBJECT DETAILS**

***Ethical Approval and Compliance***

All studies complied with the United Kingdom Home Office regulation governing the care and use of laboratory animals and with the Guide for the Care and Use of Laboratory Animals published by the US National Institutes of Health (NIH publication No. 85-23, revised 1996). All methods and protocols used in these studies were approved by the animal care and use committee of the Imperial College (London, UK) and the University of Wisconsin–Madison (USA). All animals used in this study received humane care in compliance with the Guide for the Care and Use of Laboratory Animals. Human tissue collection followed the Declaration of Helsinki and was approved by the institutional review boards of Imperial College London (REC 07/H0707/120) and the University of Verona.

***Experimental Models and Human Sample Collection***

**Sprague-Dawley Rats**

Adult Sprague-Dawley rats (Harlan Laboratories, Wyton, UK) of both sexes (>250 g) were used for either cardiomyocyte isolation or myocardial infarction surgery.

**CAG-Epac1-camps Transgenic Mice**

To monitor cAMP signalling, transgenic mice ubiquitously expressing the Epac1-camps FRET sensor under the CMV-enhancer/chicken β-actin promoter (CAG) were used (Gift from Prof. VO Nikolaev, University Medical Center Hamburg-Eppendorf, Germany) (1). The Epac1-camps construct encodes a cAMP-binding domain of Epac1 flanked by CFP and YFP (2). Genotyping was performed by PCR using validated primers, and homozygous colonies were established.

**Caveolin-3 Conditional Knockout (Cav3KO) Mice**

Inducible, cardiac-specific Caveolin-3 knockout (Cav3KO) mice were generated by crossing mice carrying loxP-flanked exon 2 of the *Cav3* gene with α-myosin heavy chain–MerCreMer (α-MHC-MerCreMer) mice (gift from Dr. Timothy Kamp, University of Wisconsin–Madison) (3). The α-MHC promoter confers cardiac specificity, while MerCreMer allows temporal control of recombination via tamoxifen induction (4, 5). Adult mice received tamoxifen (Sigma-Aldrich, T5648; 75 mg/kg/day, intraperitoneally) for five consecutive days. Mice were interbred to maintain homozygosity, and genotyping was performed by PCR to confirm recombination. Hearts were collected 21 days after the final tamoxifen injection for cardiomyocyte isolation.

**CAG-Epac1-camps/Cav3KO Double Transgenic mice**

Double transgenic mice homozygous for both the CAG-Epac1-camps construct and cardiac-specific Cav3 deletion were generated by crossing CAG-Epac1-camps mice with tamoxifen-inducible Cav3KO mice. Animals homozygous for each allele were bred for multiple generations (approximately F10) to ensure stable inheritance of both transgenes. Genotyping was performed by PCR to confirm homozygosity for *Epac1-camps* and *Cav3*-floxed alleles with *MerCreMer* expression. Expression of the Epac1-camps FRET sensor was verified using fluorescence-based screening with GFP miner goggles (Biological Laboratory Equipment, Maintenance & Service Ltd, UK) (2).

***Human Tissue***

**Patient Screening**

Human atrial tissue was obtained from patients undergoing routine cardiac surgery at Hammersmith Hospital, Imperial College London (UK), and Ospedale Civile Maggiore di Verona (Italy). Informed consent was obtained from all patients prior to tissue collection, in accordance with institutional ethical guidelines.

**Patient Groups**

Samples were collected from the left and/or right atrium during surgical procedures. A total of 22 patients were included across the following analyses: contraction studies (n = 6), FRET imaging (n = 5), and qPCR n = 11. Tissues were obtained during mitral valve (MV) or coronary artery bypass graft (CABG) surgery, as well as from heart transplant recipients with dilated (DCM) or ischaemic cardiomyopathy (ICM), including cases with and without left ventricular assist devices (LVADs).

Patients were categorised into four groups based on cardiac rhythm and left ventricular ejection fraction (LVEF). Rhythm status divided patients into sinus rhythm (SR) or atrial fibrillation (AF, including persistent and paroxysmal). LVEF stratified patients into heart failure (HF, EF < 40%) or non-HF (nHF, EF > 40%) groups.

**Patient Characteristics**

Patient Characteristic data are summarised below:

| Total number of patients | 36 |
| --- | --- |
| Age, year (Mean ± SD) | 67±8 |
| Men, n (%) | 27 (67.5%) |
| Women, n (%) | 13 (23.5%) |
| **Surgical Procedure** |  |
| \| Single Valve \| \| --- \| | \| 5 \| (12%) \| \| --- \| --- \| |
| \| Valve + Valve \| \| --- \| | \| 2 (6%) \|  \| \| --- \| --- \| |
| \| Valve + CABG \| \| --- \| | \| 5 (12%) \|  \| \| --- \| --- \| |
| \| Valve + Other \| \| --- \| | \| 9 (23%) \|  \| \| --- \| --- \| |
| \| CABG + Other \| \| --- \| | \| 2 \| (6%) \| \| --- \| --- \| |
| \| Heart transplant \| \| --- \| | \| 16 \| (41%) \| \| --- \| --- \| |
| **Previous Medical History** |  |
| Myocardial Infarction | 13 (32%) |
| Cardiac Intervention | 8 (21%) |
| Previous Cardiac Surgery | 2 (5%) |
| **Previous Medications** |  |
| \| β-blockers \|  \| \| --- \| --- \| | 9 (22.5%) |
| \| ACE inhibitors \| \| --- \| | \| 4 (10.0%) \| \| --- \| |
| \| ARBs \| \| --- \| | \| 2 (5.0%) \| \| --- \| |
| \| Diuretics \| \| --- \| | \| 10 (25.0%) \| \| --- \| |
| \| Digoxin \| \| --- \| | \| 2 (5.0%) \| \| --- \| |
| \| Calcium antagonists \| \| --- \| | \| 3 (7.5%) \| \| --- \| |
| \| Statins \| \| --- \| | \| 7 (17.5%) \| \| --- \| |
| \| Aldosterone receptor antagonists \| \| --- \| | \| 2 (5.0%) \| \| --- \| |
| \| Antiplatelets \| \| --- \| | \| 9 (22.5%) \| \| --- \| |
| Anticoagulation | 3 (7.5%) |
| Inotropic agents | 1 (2.5%) |

***Rat heart failure model***

Chronic heart failure (HF) was induced in both mice and rats by permanent ligation of the left anterior descending (LAD) coronary artery, as previously described (6, 7).

In mice, adult male C57BL/6J mice (8–10 weeks old) underwent LAD ligation at the Cardiovascular Physiology Core Facility, University of Wisconsin–Madison. Animals were anaesthetised with 2% isoflurane and ventilated with oxygen. A left thoracotomy was performed, and the LAD was ligated ~1–2 mm from its origin using an 8-0 nylon suture. Myocardial infarction (MI) was confirmed by pallor of the left ventricular (LV) wall. Mice were recovered on a heating pad and monitored daily. At 8 weeks post-MI, transthoracic echocardiography (Vevo2100, VisualSonics) was used to confirm HF, with mice showing left ventricular ejection fraction (LVEF) ≤ 40% included in downstream experiments.

In rats, adult Sprague-Dawley males (≥250 g) were anaesthetised with isoflurane (5% induction, 2% maintenance) via mechanical ventilation. Animals received enrofloxacin (5 mg/kg) and sterile saline (10 mL/kg) pre-operatively, and buprenorphine (0.05 mg/kg) subcutaneously for analgesia. A left thoracotomy was performed at the fourth intercostal space, and following pericardiotomy, the LAD was ligated ~2 mm below its origin using a 6-0 silk suture (Ethicon) to induce anterior MI. Postoperative care followed institutional guidelines, and animals were housed under standard conditions. Atrial cardiomyocytes were isolated 16 weeks post-ligation for functional analyses.

***Atrial Cardiomyocyte Isolation from Rodents (Rats and Mice)***

**Rat Atrial Cardiomyocyte Isolation:**

Rats were anaesthetised with 5% isoflurane in 95% O₂ and euthanised via cervical dislocation. Hearts were rapidly excised and transferred to ice-cold, oxygenated Krebs-Henseleit (KH) solution containing 119 mM NaCl, 4.7 mM KCl, 0.94 mM MgSO₄, 1.2 mM KH₂PO₄, 25 mM NaHCO₃, 11.5 mM glucose, and 1 mM CaCl₂, continuously bubbled with 95% O₂ / 5% CO₂. After trimming excess tissue, the heart was cannulated via the aorta and mounted on a Langendorff apparatus.

Perfusion was initiated with KH buffer for 5 minutes at 37°C, followed by 5 minutes of a calcium-free solution (12–15 μM CaCl₂, 120 mM NaCl, 5.4 mM KCl, 5 mM MgSO₄, 5 mM pyruvate, 20 mM glucose, 20 mM taurine, 10 mM HEPES, 5 mM nitrilotriacetic acid (NTA); pH 7.4, 100% O₂). Enzymatic digestion was carried out in two phases: (1) perfusion with protease type XXIV (3.8 mg/mL, Sigma) in low-Ca²⁺ buffer with Ca²⁺ adjusted to 150 μM for 20 minutes; and (2) mechanical dissection of the atria followed by incubation in collagenase type V (1 mg/mL, Sigma) at 37°C for 6 minutes with gentle shaking. The digested tissue was passed through a 200 μm nylon mesh and cells were stored in enzyme-free buffer at room temperature until use.

**Mouse Atrial Cardiomyocyte Isolation:**

Adult mice were anaesthetised and euthanised similarly. The heart was excised, cannulated, and mounted on a Langendorff apparatus. Following 5 minutes of KH perfusion at 37°C, the heart was perfused with calcium-free buffer for another 5 minutes. Enzymatic digestion involved perfusion with protease type XXIV (3.8 mg/mL) for 4–6 minutes (depending on heart size), followed by collagenase type V (1 mg/mL) digestion after atrial dissection for an additional 5–6 minutes at 37°C. The cell suspension was filtered through a 200 μm mesh and left in enzyme-free buffer at room temperature.

For both species, only rod-shaped, non-contracting cardiomyocytes with clear cross-striations were selected for experiments. Cell viability and morphology were verified via phase-contrast microscopy.

**Human atrial cardiomyocyte isolation**

Human cardiomyocytes were isolated from atrial biopsies by enzymatic digestion as described before(8). Atrial cardiomyocytes were enzymatically isolated from biopsy samples obtained during cardiac surgery. Fresh tissue was immediately immersed in cold, oxygenated low-calcium storage buffer (pH 6.95) containing 120 mM NaCl, 5.4 mM KCl, 5 mM MgSO₄, 12 μM CaCl₂, 20 mM glucose, 10 mM HEPES, 5 mM nitrilotriacetic acid (NTA), 20 mM taurine, and 5 mM pyruvate. Samples were finely minced into ~1–2 mm³ fragments, rinsed with fresh oxygenated low-Ca²⁺ solution, and gently agitated three times for 3 minutes to remove residual blood.

Initial enzymatic digestion was performed in enzyme buffer supplemented with protease type XXIV (0.36 mg/mL, Sigma) and collagenase type V (0.5 mg/mL, Sigma) for 10–15 minutes at 37°C with gentle shaking. The partially digested tissue was transferred to fresh enzyme buffer containing collagenase type XIV (1 mg/mL, Sigma) for up to three repeated 10-minute incubations. Following enzymatic digestion, the suspension was filtered through a 200 μm nylon mesh and centrifuged at 600 rpm for 3 minutes. The supernatant was removed and the resulting cell pellet was re-suspended in fresh enzyme-free buffer and maintained at room temperature until use.

***IonOptix-based measurements of cell contractility***

Contractility was assessed by measuring sarcomere shortening in isolated atrial cardiomyocytes using an IonOptix system (IonOptix, USA). Cells were placed in a custom chamber on an inverted microscope and continuously perfused with warmed KH solution (119 mM NaCl, 4.7 mM KCl, 0.94 mM MgSO₄, 1.2 mM KH₂PO₄, 25 mM NaHCO₃, 11.5 mM glucose, 1 mM CaCl₂, gassed with 95% O₂ / 5% CO₂) maintained at 37°C via a peristaltic pump.

Cells were field-stimulated using platinum electrodes delivering suprathreshold square-wave pulses (50 V): 0.2 Hz for human, 0.5 Hz for rat, and 1 Hz for mouse atrial myocytes. After a baseline period, β₂-adrenergic receptors were selectively blocked using ICI 118,551 (50 nM, Tocris) for 10 minutes. β₁-adrenergic stimulation was then achieved by perfusion with isoproterenol (ISO; 100 nM, Sigma). Finally, A1 adenosine receptor activation was induced using the selective agonist 2-chloro-N(6)-cyclopentyladenosine (2-MeCCPA; 1 μM, Tocris).

Cell shortening was calculated as percentage change in sarcomere length across ten consecutive contractions at each treatment phase, with data analysed using IonOptix software (IonWizard).

***FRET-based measurements of intracellular cAMP dynamics***

Intracellular cAMP dynamics were measured using an Epac-based FRET biosensor (RII_Epac; gift from Prof. M. Zaccolo, University of Oxford), expressed via adenoviral transduction in isolated rat and human atrial cardiomyocytes. Cells were cultured for ≥48 h on laminin-coated MatTek dishes or coverslips in Medium 199 (Thermo Fisher) supplemented with 5 mM creatine, 5 mM taurine, 5 mM carnitine, 1 g/L BSA, 100 μM ascorbate, and 1× penicillin/streptomycin.

FRET imaging was performed on a Nikon TE2000 inverted microscope equipped with a 60×/1.40 NA oil immersion objective, a DualView beam splitter (Photometrics, D535/40 and D430/30 emission filters), and an ORCA-ER CCD camera (Hamamatsu, UK). Fluorescence signals were recorded using Micro-Manager 1.4 software. β₂ARs were inhibited with ICI 118,551 (100 nM), followed by βAR stimulation with isoproterenol (100 nM) to stimulate β₁AR, A1AR activation with 2-MeCCPA (1 μM), and non-specific PDE inhibition with IBMX (100 μM, Sigma I5879). YFP and CFP signals were corrected for bleed through and used to calculate FRET ratios (YFP/CFP) with a custom ImageJ plugin.

***Combined FRET/SICM measurements of local cAMP signaling***

Localised cAMP signalling responses in cardiomyocyte microdomains were measured by combining Förster resonance energy transfer (FRET) imaging with scanning ion conductance microscopy (SICM). Cardiomyocytes expressing the RII_Epac biosensor were plated on laminin-coated MatTek dishes. Cells were imaged using SICM equipped with nanopipettes (inner diameter approximately 100 nm). High-resolution topographical scans (5 µm × 5 µm areas) of cardiomyocyte surfaces were acquired to identify structural features including transverse tubule (T-tubule) openings and surface crest regions, as previously described (9).

Cells were initially treated selective β₂AR antagonist ICI 118,551 (50 nM; Tocris) to isolate β₁AR -mediated signalling. Whole cell β₁AR activation was induced by bath application of isoproterenol (100 nM, Sigma), and the global FRET response was continuously monitored until reaching a stable plateau.

Localised stimulation of A1AR was performed by delivering the selective agonist 2-MeCCPA (1 µM; Tocris) via the scanning nanopipette, using voltage-controlled application (from +500 mV holding potential to −1,000 mV). Agonist was applied directly into identified T-tubule openings or onto crest membrane regions, guided by SICM topography coordinates. Localised FRET responses were simultaneously recorded using Micro-Manager 1.4 software. FRET data were analysed using a custom ImageJ plugin, as described above.

***Super-resolution Scanning Patch-Clamp for LTCC Activity***

Single-channel recordings of LTCC were performed using a combined SICM and patch-clamp approach as previously described(10). Topographical surface images of cardiomyocytes were first obtained using SICM system with nanopipettes (initial resistance ~100 MΩ, 10 µm × 10 µm areas). Subsequently, pipette opening was widened by carefully clipping the tip to an average resistance of 30 MΩ, enabling stable cell-attached patch-clamp recordings. Targeted positioning of pipettes at either T-tubule or crest regions was guided by SICM imaging.

Recordings were performed at room temperature in cell-attached configuration using an Axopatch 200A amplifier (Axon Instruments, USA) controlled by pClamp software (version 10, Axon Instruments). The external bath solution contained (in mM): 120 K-gluconate, 25 KCl, 2 MgCl₂, 1 CaCl₂, 2 EGTA, 10 glucose, and 10 HEPES (pH 7.4 adjusted with KOH). The pipette solution comprised (in mM): 90 BaCl₂, 10 HEPES, and 10 sucrose (pH 7.4 adjusted with TEA-OH). Liquid junction potential was calculated to be -16.7 mV and corrected in all data.

Single-channel currents were sampled at 10 kHz, filtered at 2 kHz using an 8-pole Bessel filter, and analyzed offline with Clampfit software (version 10.2, Axon Instruments). The Po was averaged from 50 sweeps at –6.7 mV for each cell.

***Optical mapping of calcium transients***

Calcium transients (CaTs) were measured in isolated mouse atrial tissue preparations and single isolated rat atrial cardiomyocytes using optical mapping as previously described(8, 11). Mouse atrial tissues were loaded with the fluorescent calcium indicator Rhod-2 AM (10 µM; Invitrogen, USA) for 30–45 min at 37°C, followed by washout and incubation with the excitation-contraction uncoupler blebbistatin (10–20 µM; Tocris, UK) to minimise motion artefacts during imaging. Imaging was performed using a MiCAM Ultima-L high-speed CMOS camera system (SciMedia, USA) at a spatial resolution of 100 µm/pixel and temporal resolution ranging from 500 to 1000 frames per second. Illumination for excitation was provided by an LED source with excitation/emission filters appropriate for Rhod-2 fluorescence (excitation: 540 ± 25 nm, emission: >590 nm).

For isolated rat atrial cardiomyocytes, cells were plated onto MatTek dishes and loaded with Fluo-4 AM (10 µM; Invitrogen, USA) in a KH buffer at 37°C for 30 min, followed by washout. Single-cell imaging was performed on an inverted Nikon Eclipse Ti microscope fitted with the MiCAM Ultima-L camera system at a spatial resolution of 1 µm/pixel and temporal resolution of 500 to 1000 frames per second.

***Quantitative Real-time PCR (qRT-PCR)***

Total RNA was extracted from atrial tissue samples using the peqGOLD Total RNA Isolation Kit (Peqlab, Germany) following the manufacturer’s instructions. RNA purity and concentration were assessed using a NanoDrop 2000 Spectrophotometer (Thermo Fisher Scientific, USA).

Complementary DNA (cDNA) was synthesised from 1 µg of total RNA using the High-Capacity cDNA Reverse Transcription Kit (Applied Biosystems, USA) according to the manufacturer’s protocol. Quantitative real-time PCR (qRT-PCR) was performed using SYBR® Green JumpStart™ Taq ReadyMix™ (Sigma-Aldrich, UK) in an Eppendorf Mastercycler ep realplex Real-Time PCR System (Eppendorf, Germany).

Reaction conditions were as follows: initial denaturation at 95°C for 10 minutes, followed by 40 cycles of denaturation at 95°C for 15 seconds and annealing/extension at 60°C for 60 seconds. Gene-specific primer sequences used in this study are listed in Supplementary Table 1.

Relative gene expression was calculated using the comparative cycle threshold (Ct) method (2^−ΔΔCt), with mRNA levels normalised to a validated internal reference gene (housekeeping gene; GAPDH). Stability and suitability of GAPDH as a reference gene were confirmed by verifying minimal variability in Ct values across experimental conditions. All samples were analysed in triplicate, and data are presented as fold changes relative to control groups.

**Supplementary table 1.** Oligonucleotides used for real-time PCR for rat genes

| **Target gene** |  | **Primer sequence** |
| --- | --- | --- |
| *A_1_AR* (**[NM_017155.2](https://www.ncbi.nlm.nih.gov/nuccore/NM_017155.2)**) | Fw | 5´- GAGCTGAAGATCGCCAAGTCG-3´ |
|  | Rv | 5´- TGGGAGGTCTTCATCGATGGG-3´ |
| *A_2a_AR* ([**NM_001357942.1**](https://www.ncbi.nlm.nih.gov/nuccore/NM_001357942.1)) | Fw | 5´- CCATGCTGGGCTGGAACA -3´ |
|  | Rv | 5´- GAAGGGGCAGTAACACGAACG -3´ |
| *A_3_AR* (**[NM_001302755.1](https://www.ncbi.nlm.nih.gov/nuccore/NM_001302755.1)**) | Fw | 5´- TCTTCACCCACGCTTCCATC -3´ |
|  | Rv | 5´- GGTCAGTCCCACCAGAAAGG -3´ |
| *Rpl32* ([**NM_013226.2**](https://www.ncbi.nlm.nih.gov/nuccore/NM_013226.2)**)** | Fw | 5´- TCTGGTCCACAATGTCAAGG-3´ |
|  | Rv | 5´- TGTGCTGCTCTTTCTACGATG-3´ |

***Transmission Electron Microscopy***

Transmission electron microscopy was performed as previously described(12-15). Mouse atrial tissue was isolated as previously described and fixed in the following fixative: 2.5% glutaraldehyde, 2.0% paraformaldehyde, and 0.2 mol/l cacodylate buffer for 24 - 48 hours(12, 14). The samples were rinsed in the same buffer, post-fixed in 1% osmium tetroxide, dehydrated in a graded ethanol series, rinsed in propylene oxide, and embedded in Epon 812 substitute. After resin polymerization, the samples were then sliced into 70-nm sections with a Leica EM UC6 ultramicrotome and placed on 200 mesh transmission electron microscopy grids. The samples were post-stained in 8% uranyl acetate in 50% EtOH and Reynold's lead citrate, viewed on a Philips CM120 transmission electron microscope, and documented with a SIS MegaView III digital camera. Electron microscopy images were analyzed by using the NIH ImageJ software. A threshold size for individual caveolae was set between 50 and 100 nm. The number of caveolae was counted as per unit length (μm) of myocyte sarcolemmal membranes from a series of random electron microscopy micrographs.

***Quantification and statistical analysis***

Data analysis and graphical presentation were performed using GraphPad Prism (version 5; GraphPad Software, USA) and OriginPro (version 6.1; OriginLab Corporation, USA). All data are expressed as mean ± standard error of the mean (SEM). Normality of data distribution was assessed using the Kolmogorov–Smirnov test. For normally distributed datasets, comparisons between two groups were conducted using unpaired Student’s *t*-test, and multiple-group comparisons were analysed by one-way analysis of variance (ANOVA) followed by Tukey’s or Bonferroni’s post hoc tests, as appropriate. When datasets did not conform to normality, the non-parametric Mann–Whitney test was applied for two-group comparisons, and the Kruskal–Wallis test with Dunn’s multiple comparison post hoc test was used for comparisons among more than two groups. Categorical data were evaluated using either the Chi-square or Fisher’s exact test, as appropriate. Differences were considered statistically significant at *P* < 0.05.

***References***

1. Nikolaev VO, Bünemann M, Hein L, Hannawacker A, Lohse MJ. Novel single chain cAMP sensors for receptor-induced signal propagation. J Biol Chem. 2004;279(36):37215-8.

2. Calebiro D, Nikolaev VO, Gagliani MC, de Filippis T, Dees C, Tacchetti C, et al. Persistent cAMP-signals triggered by internalized G-protein-coupled receptors. PLoS Biol. 2009;7(8):e1000172.

3. Sohal DS, Nghiem M, Crackower MA, Witt SA, Kimball TR, Tymitz KM, et al. Temporally regulated and tissue-specific gene manipulations in the adult and embryonic heart using a tamoxifen-inducible Cre protein. Circ Res. 2001;89(1):20-5.

4. Markandeya YS, Feng L, Ramchandran V, Vaidyanathan R, Best J, Lea ML, et al. Abstract 19749: Cardiac-specific Deletion of Caveolin-3 Delays Repolarization and Increases Susceptibility to Ventricular Arrhythmia. Circulation. 2015;132(suppl_3):A19749-A.

5. Wright PT, Bhogal NK, Diakonov I, Pannell LMK, Perera RK, Bork NI, et al. Cardiomyocyte Membrane Structure and cAMP Compartmentation Produce Anatomical Variation in β(2)AR-cAMP Responsiveness in Murine Hearts. Cell Rep. 2018;23(2):459-69.

6. Lyon AR, MacLeod KT, Zhang Y, Garcia E, Kanda GK, Lab MJ, et al. Loss of T-tubules and other changes to surface topography in ventricular myocytes from failing human and rat heart. Proc Natl Acad Sci U S A. 2009;106(16):6854-9.

7. Lang D, Ni H, Medvedev RY, Liu F, Alvarez-Baron CP, Tyan L, et al. Caveolar Compartmentalization of Pacemaker Signaling is Required for Stable Rhythmicity of Sinus Nodal Cells and is Disrupted in Heart Failure. bioRxiv. 2024:2024.04.14.589457.

8. Glukhov AV, Balycheva M, Sanchez-Alonso JL, Ilkan Z, Alvarez-Laviada A, Bhogal N, et al. Direct Evidence for Microdomain-Specific Localization and Remodeling of Functional L-Type Calcium Channels in Rat and Human Atrial Myocytes. Circulation. 2015;132(25):2372-84.

9. Nikolaev VO, Moshkov A, Lyon AR, Miragoli M, Novak P, Paur H, et al. Beta2-adrenergic receptor redistribution in heart failure changes cAMP compartmentation. Science. 2010;327(5973):1653-7.

10. Bhargava A, Lin X, Novak P, Mehta K, Korchev Y, Delmar M, et al. Super-resolution scanning patch clamp reveals clustering of functional ion channels in adult ventricular myocyte. Circ Res. 2013;112(8):1112-20.

11. Lang D, Glukhov AV. High-resolution Optical Mapping of the Mouse Sino-atrial Node. J Vis Exp. 2016(118).

12. Lang D, Ni H, Medvedev RY, Liu F, Alvarez-Baron CP, Tyan L, et al. Caveolar Compartmentalization of Pacemaker Signaling is Required for Stable Rhythmicity of Sinus Nodal Cells and is Disrupted in Heart Failure. bioRxiv. 2024.

13. Turner DGP, De Lange WJ, Zhu Y, Coe CL, Simcox J, Ge Y, et al. Neutral sphingomyelinase regulates mechanotransduction in human engineered cardiac tissues and mouse hearts. J Physiol. 2024;602(18):4387-407.

14. Medvedev RY, Turner DGP, DeGuire FC, Leonov V, Lang D, Gorelik J, et al. Caveolae-associated cAMP/Ca(2+)-mediated mechano-chemical signal transduction in mouse atrial myocytes. J Mol Cell Cardiol. 2023;184:75-87.

15. Egorov YV, Lang D, Tyan L, Turner D, Lim E, Piro ZD, et al. Caveolae-Mediated Activation of Mechanosensitive Chloride Channels in Pulmonary Veins Triggers Atrial Arrhythmogenesis. J Am Heart Assoc. 2019;8(20):e012748.


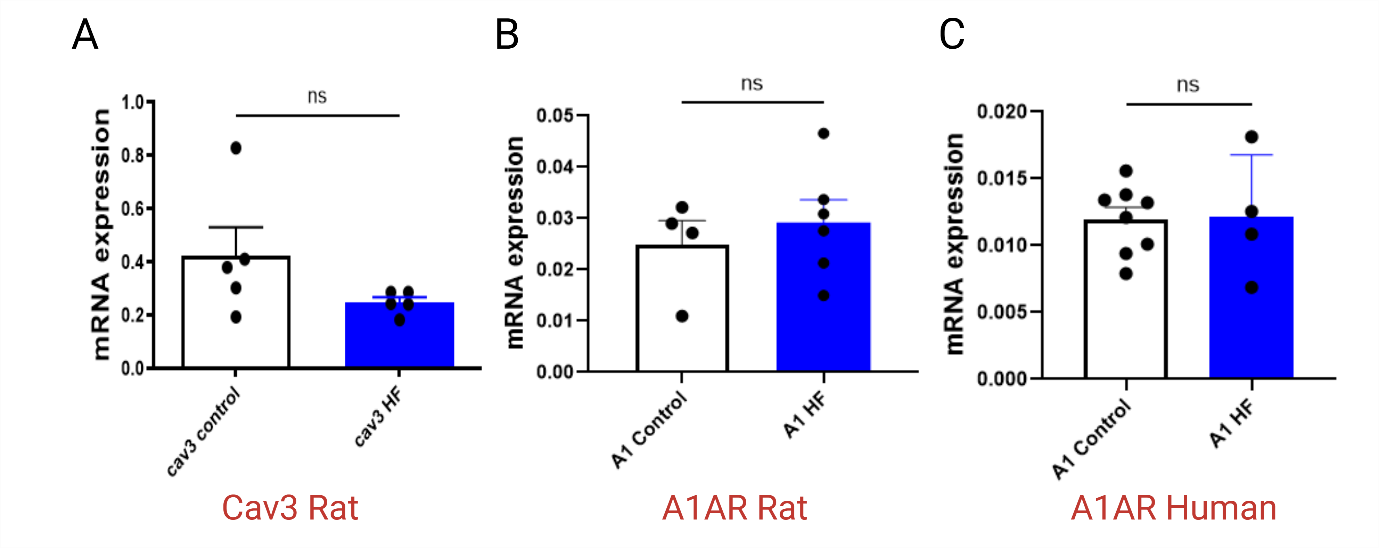


**Supplementary Figure 1. Cav3 and A1AR expression levels are preserved in right atrial tissue in human and rat heart failure.** Quantitative real-time PCR analysis of Caveolin-3 (Cav3) mRNA expression in rat atrial tissue (A) and A1 adenosine receptor (A1AR) mRNA expression in rat (B) and human (C) atrial samples from control and heart failure (HF) groups. Gene expression was normalised to GAPDH and calculated using the ΔΔCt method. No significant differences in Cav3 or A1AR transcript levels were detected between control and HF conditions in either species. (A: N=5 Rats per group; B: N=4-6 Rats per group; C: N= 4-8 Patients per group). Data are shown as individual values with mean ± SEM; ns, not significant.


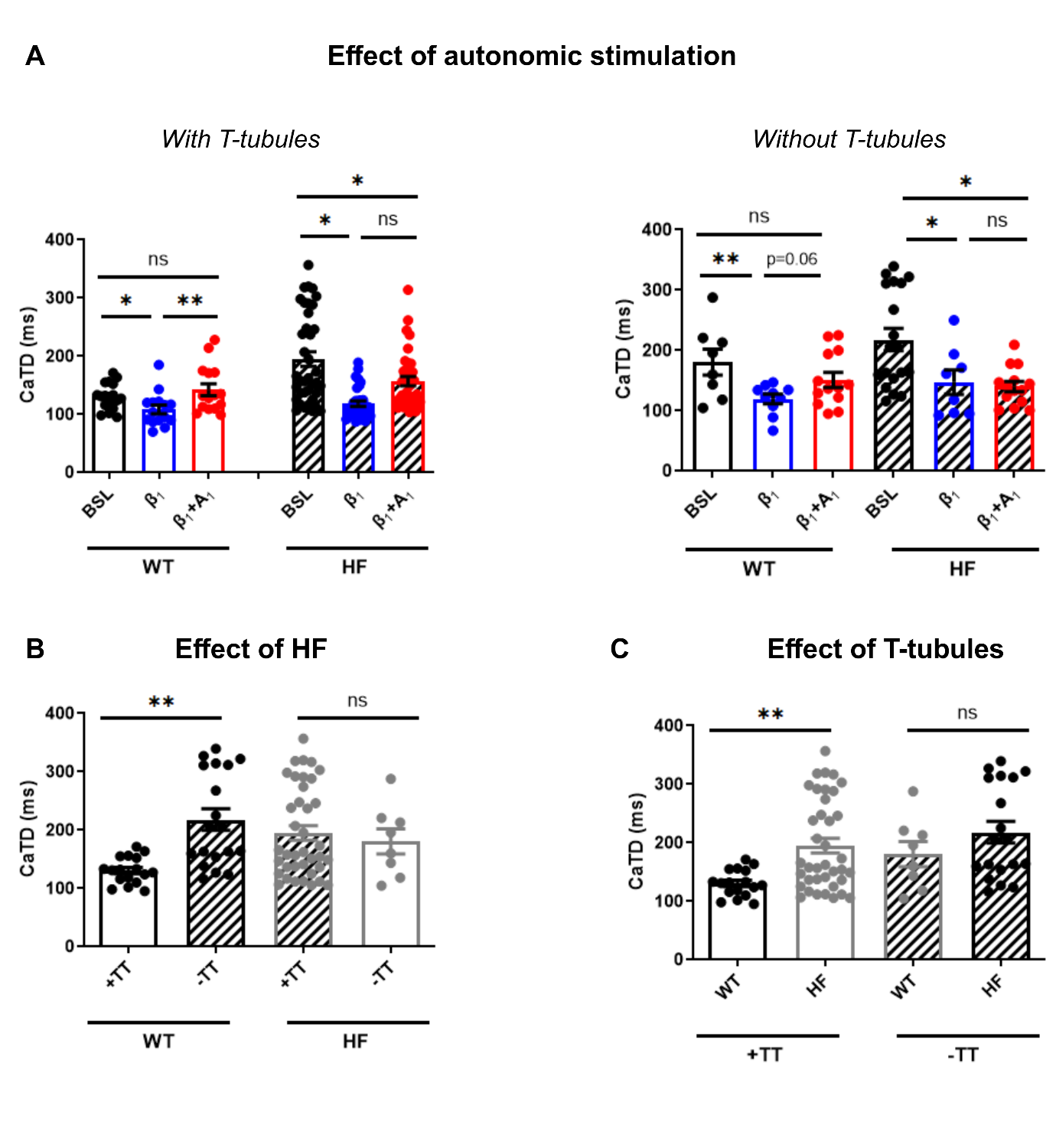
**Supplementary Figure 2. A_1_AR anti-adrenergic effects on calcium transient duration (CaTD) are dependent on T-tubule structure and selectively impaired in heart failure. (A)** Effect of autonomic stimulation on CaTD in atrial myocytes with (*left*) and without (*right*) T-tubules from wild-type (WT) and heart failure (HF) rats. CaTD was measured at baseline (BSL), following β₁-adrenergic receptor (β₁AR) stimulation (β₁), and after subsequent adenosine A_1_ receptor (A_1_AR) stimulation (β₁ + A_1_). In WT myocytes with T-tubules, A_1_AR stimulation significantly reversed β₁AR-induced CaTD shortening (P = 0.01). A partial, non-significant reversal was observed in WT myocytes without T-tubules (P = 0.06). In HF myocytes, A_1_AR stimulation did not reverse β₁AR-induced CaTD shortening in either T-tubule-containing (NS) or T-tubule-deficient cells (NS). **(B)** Effect of heart failure on baseline CaTD. Baseline CaTD was significantly prolonged in T-tubule-containing myocytes in HF compared to WT (P = 0.01). No difference was observed in T-tubule-deficient myocytes between groups (NS). **(C)** Effect of T-tubule structure on baseline CaTD. In WT myocytes, CaTD was significantly longer in T-tubule-deficient cells compared to T-tubule-containing cells (P = 0.002). This difference was abolished in HF (NS). Data are presented as box-and-whisker plots indicating median, interquartile range, and full data spread. P-values indicate statistical significance as determined by appropriate comparisons.
